# The influence of risk perceptions on close contact frequency during the SARS-CoV-2 pandemic

**DOI:** 10.1038/s41598-022-09037-8

**Published:** 2022-03-25

**Authors:** James Wambua, Lisa Hermans, Pietro Coletti, Frederik Verelst, Lander Willem, Christopher I. Jarvis, Amy Gimma, Kerry L. M. Wong, Adrien Lajot, Stefaan Demarest, W. John Edmunds, Christel Faes, Philippe Beutels, Niel Hens

**Affiliations:** 1 UHasselt, Data Science Institute, I-BioStat, 3500 Hasselt, Belgium; 2 Centre for Health Economic Research and Modelling Infectious Diseases, Vaccine and Infectious Disease Institute, University of Antwerp, 2610 Antwerp, Belgium; 3 Department of Infectious Disease Epidemiology, Centre for Mathematical Modelling of Infectious Diseases, London School of Hygiene and Tropical Medicine, Keppel Street, London, WC1E 7HT UK; 4 Department of Epidemiology and Public Health, Sciensano, Brussels, Belgium; 5 School of Public Health and Community Medicine, The University of New South Wales, Sydney, NSW 2033 Australia

**Keywords:** Medical research, Epidemiology, Health care, Public health, Epidemiology

## Abstract

Human behaviour is known to be crucial in the propagation of infectious diseases through respiratory or close-contact routes like the current SARS-CoV-2 virus. Intervention measures implemented to curb the spread of the virus mainly aim at limiting the number of close contacts, until vaccine roll-out is complete. Our main objective was to assess the relationships between SARS-CoV-2 perceptions and social contact behaviour in Belgium. Understanding these relationships is crucial to maximize interventions’ effectiveness, e.g. by tailoring public health communication campaigns. In this study, we surveyed a representative sample of adults in Belgium in two longitudinal surveys (survey 1 in April 2020 to August 2020, and survey 2 in November 2020 to April 2021). Generalized linear mixed effects models were used to analyse the two surveys. Participants with low and neutral perceptions on perceived severity made a significantly higher number of social contacts as compared to participants with high levels of perceived severity after controlling for other variables. Our results highlight the key role of perceived severity on social contact behaviour during a pandemic. Nevertheless, additional research is required to investigate the impact of public health communication on severity of COVID-19 in terms of changes in social contact behaviour.

## Introduction

Since the emergence of the new coronavirus SARS-CoV-2, more than 435 million cases and 5.9 million deaths have been reported globally as of March, 7th 2022^1^. Due to the unprecedented high number of deaths and its global spread, the pandemic has had more far reaching negative health and socio-economic implications as compared to previous pandemics^2,3^. Before COVID-19 vaccines became available, many governments across the globe focused on emphasizing and implementing several Non-Pharmaceutical Interventions (NPIs) such as hand hygiene, mask wearing and social distancing measures to curb the spread of the virus. Even though vaccination is now considered a priority in halting the pandemic, NPIs remain an important part of policymakers’ strategies until a sufficient level of immunity has been reached on a global scale. Many of these NPIs have previously been implemented only in limited regional settings and for limited time periods in previous pandemics such as during the 2002–2004 SARS-CoV-1 epidemic^4,5^ and the influenza A(H1N1)v2009 pandemic^6^. Thus, a considerable proportion of individuals in the population lacks prior experience in responding to pandemics^7^. Since the adoption of preventive measures can mostly be implemented at the individual-level, substantial differences in compliance might be inherently due to varying demographic and attitudinal determinants^8^.

Since we are in the midst of a crisis, empirical research findings on the key attitudinal factors modulating behavioral responses are of great relevance in order to enable implementation of tailored strategies. These findings are also important to enhance continued formulation of both socially and economically acceptable policies that can aid in the short and also long-term management of the COVID-19 pandemic. Furthermore, given the prolonged nature of the pandemic, which coincided with changing regimes of intervention measures, we might expect evolution of both perceptions and protective behaviours, as was observed during the A(H1N1)v2009 pandemic^9,10^.

Many empirical studies have assessed the relationships between the public perceptions and the adoption of protective behaviours for COVID-19 and other emerging infectious diseases like A(H1N1)v2009 and SARS-CoV-1^6–9,11–22^. The two most common theories in which these studies have been based are the Health Belief Model (HBM) and the Protection Motivation and Self-efficacy theory (PMS). According to the HBM, individuals are likely to implement health protective behaviours based on how they perceive themselves to be at risk^23^. The PMS on the other hand emphasizes that individuals tend to adapt to recommended health preventive measures according to how effective they perceive the measures to be, and also according to how they believe they are capable to adhere to them^24^.

Several studies have examined the role of risk perceptions in the adoption of the recommended protective measures. In particular, perceived severity if infected has been found to be associated with adoption of protective behaviors^6–9,12,15,21,22^ in line with the HBM. Other studies have explored and found associations between public health belief in the effectiveness of intervention measures and uptake of protective behaviours^4–6,15^. While others have found that confidence to adhere to the imposed measures was related to the actual performance of the measures^4,25,26^ which is in line with the PMS theory.

Human behaviour is known to be crucial in the propagation of infectious diseases that are spread through respiratory or close-contact routes such as the SARS-CoV-2 virus^27,28^. Therefore, intervention measures implemented to curb the spread of the virus mainly aim at limiting the number of close contacts. However, studies explicitly exploring the relationships between perceptions and contact behaviour in pandemic times are lacking in the literature. Thus, given the importance of contact behaviour in the transmission dynamics of infectious diseases^27–30^, it is crucial to monitor social contact behaviour in relation to changes in specific COVID-19 perceptions for the continued management of the crisis.

This paper’s main objective is to explore the relationship between COVID-19 related perceptions and the number of social contacts using data from the CoMix study, a longitudinal survey in which individuals are asked about their attitudes, awareness, and behaviours in response to COVID-19 over time in Europe^31^. We use data from two longitudinal surveys involving panels of participants in Belgium. The first survey involved 8 waves of data collection between April 2020 and August 2020^30^. The second survey is ongoing and we report results of the first 11 survey waves between November 2020 and April 2021^31^. The survey panels were representative with respect to gender, age and region of residence.

## Results

### Sample composition and GLMM summary results

Summary of the sample composition in the first and second analysis are contained in Supplementary Tables S1 and S2, respectively. The average number of contacts in the first and second analysis are contained in Table 2 in Coletti et al.^30^ and Supplementary Table S3, respectively. The average number of waves in which participants participated in the study in the first and second analysis was 4.97 and 4.87, respectively. Results from the GLMM indicated that the overdispersion parameter ranged between 2.47 (95% CI 2.29–2.67) and 2.54 (95% CI 2.35–2.74) in the first analysis, whilst in the second analysis, this parameter ranged between 1.82 (95% CI 1.71–1.92) and 1.85 (95% CI 1.74–1.96) indicating substantial heterogeneity. The variance of the random effect ranged between 0.438 and 0.447 in the first analysis from the different individual perception models while in the second analysis, it ranged between 0.663 and 0.700. In both analyses, the random effect was statistically significant (p-value $< 0.001$), further indicating underlying heterogeneity in the social contact behaviour among individuals.

### Perceived severity

Results from the first analysis in the GLMM model indicated that in the perceived severity model, participants with low and neutral level of perceived severity made 1.70 (95% CI 1.18–2.45) and 1.56 (95% CI 1.14–2.14) times more contacts than the participants with high levels of perceived severity, respectively (Supplementary Table S4). The predicted number of contacts for the high level of perceived severity was 1.41 (95% CI 1.10–1.79), while for the low and neutral levels was 2.38 (95% CI 1.69–3.36) and 2.20 (95% CI 1.61–3.00), respectively. In the second analysis, participants with low and neutral levels of perceived severity made 1.62 (95% CI 1.14–2.31) and 1.76 (95% CI 1.31–2.38) times more contacts than the participants with high levels of perceived severity, respectively (Supplementary Table S5). The predicted number of contacts for the high level was 1.42 (95% CI 1.05–1.91) whilst for the low and neutral levels was 2.31 (95% CI 1.67–3.18) and 2.51 (95% CI 1.89–3.33), respectively.

The interaction term between perceived severity and survey wave of the data collection was significantly associated with the number of contacts in both analyses from the Type III Wald tests (p-values = 0.002 and < 0.001, respectively). Participants with high levels of perceived severity had fewer social contacts as compared to those with low or neutral perceived severity (Fig. 3). Marginal effects of the interaction terms between perceived severity and age group depicted similar patterns in both analyses (Fig. 4). The interaction term between perceived severity and participants’ age group was significantly associated with the number of contacts in the second analysis only (11 waves) (p-value = 0.039). For more information on the significance of the variables in both analyses for perceived severity, see Supplementary Table S6. The interaction effects between the perceived severity and age in the first analysis were more pronounced in the youngest age group [18,30) where large differences in the predicted number of contacts were observed between participants with high level of perceived severity and those with low or neutral perceptions. Small differences in predicted number of contacts were observed in age groups [30,40) and 70+ among the different levels of perceived severity. In the second analysis, the interaction effects similarly showed more pronounced differences in the predicted number of contacts among the different perceived severity levels in the age group [18,30) as compared to the other age groups.

### Perceived susceptibility

The GLMM results from the perceived susceptibility model indicated that the interaction terms between perceived susceptibility and age group and between perceived susceptibility and wave of data collection were significantly associated with the number of contacts in both analyses (p-values 0.025; 0.038 and <0.001; <0.001, respectively). Furthermore, the GLMM results from the first analysis indicated significant interaction effects between perceived susceptibility and face mask wearing and between perceived susceptibility and household size (p-values = 0.05 and 0.014, respectively) see (Supplementary Table S7). In the first analysis, the number of contacts was found to be higher for participants with low levels of perceived susceptibility in younger age groups ([18,30) and [30,40)), and generally higher for those with high levels of perceived susceptibility in the other age groups. Whilst in the second analysis, participants with high levels of perceived susceptibility generally made a higher number of contacts than those with low and neutral perceptions (Supplementary Fig. S1). Similarly, plots of the predicted number of contacts from the marginal effects of the interaction terms between perceived susceptibility and wave of data collection in both the first and second analyses were also slightly different. In the first analysis, participants with low levels of perceived susceptibility had a higher number of contacts. While this was not the case in the second analysis where participants with neutral perceptions of susceptibility had a higher number of contacts (Supplementary Fig. S2). Plots of the predicted number of contacts from the marginal effects of the interaction terms between perceived susceptibility and face mask wearing indicated that participants who had low levels of perceived susceptibility made more number of contacts than those who had neutral or high levels. Furthermore, we observed that participants who indicated to have used a face mask made more number of contacts (Supplementary Fig. S3). The interaction effects between perceived susceptibility and household size indicated that an increase in household size coincided with an increasing trend in the predicted number of contacts for all the levels of perceived susceptibility (Supplementary Fig. S3).

### Perceived benefit to vulnerable

In both analyses, the interaction term between perceived benefit to vulnerable and wave of data collection was significant (all p-values <0.05), see Supplementary Table S8. However, we did not observe any distinct pattern in terms of the number of contacts among the different perception levels (Supplementary Fig. S4).

### Perceived effectiveness of measures

The perceived effectiveness of measures was only considered in the first analysis. We found a significant interaction effect between the perceived effectiveness of measures and wave of data collection (p-value = 0.013) in the perceived effectiveness model in the first analysis. Plots of the predicted number of contacts from the marginal effect of the interaction term between perceived effectiveness and wave of data collection showed that participants with high levels of perceived effectiveness generally made fewer contacts than those with low levels of perceived effectiveness (Supplementary Fig. S5). However, the observed differences were small.

### Perceived adherence to measures

Similarly, the perceived adherence to measures was considered in the first analysis only. We observed a significant interaction effect between perceived adherence to measures and participant’s age group (p-value = 0.015). The plots of the predicted number of contacts from the marginal effects of the interaction term between perceived adherence to measures and participant’s age group showed that in general, participants with high levels of perceived adherence made fewer contacts than those with low levels of perceived adherence to measures (Supplementary Fig. S6). Similarly, the observed differences in the number of contacts were small.

Descriptive plots for the aforementioned COVID-19 related perceptions and the average number of contacts in both the first and second survey rounds showed changes over time and slight variations over age groups (Supplementary Figs. S7–S11). Furthermore, the dynamics of the perceived severity and average number of reported hospitalizations tracked each other relatively well, implying the perception on severity changed with actual risk proxied by the number of new hospital admissions (Supplementary Fig. S12). In all the models considered in both the first and second analyses, we observed that household size, wave of data collection, face mask wearing and participants occupation were significant predictors of the number of social contacts in addition to the different perception variables. For more information, see (Supplementary Tables S6–S9). The GLMM results indicated that participants who reported to have used a face mask made significantly more number of contacts than those who had not used. Further, the results indicated that a one unit increase in household size coincided with 1.25 (95% CI 1.21–1.13) and 1.45 (95% CI 1.38–1.55) increase in the number of contacts from the first and second analyses, respectively (Supplementary Tables S4–S5).

## Discussion

In the face of the COVID-19 pandemic that has led to unprecedented negative health outcomes and social economic burden^2,3^, it is important to understand factors that influence individual behaviour. Our study explored the relationship between 5 specific perception variables related to COVID-19 and behavioral response in terms of the reported number of social contacts. We used a generalized linear mixed effects model in order to take into account both the within-participant and between participant variability from the two longitudinal datasets.

The results indicated that individuals who perceived themselves to experience severe illness if they contract a COVID-19 infection tended to make significantly fewer contacts as compared to those who had low or neutral perceptions. The observed relationship between the perceived severity and social contact behaviour was consistent in both analyses (i.e, analyses involving survey data from the first 8 waves of data collection, and also from the subsequent 11 waves). It is important to note that these two longitudinal surveys queried respondents’ behavior in two different COVID-19 pandemic waves in Belgium, with the first survey coinciding with the first COVID-19 wave, and the second survey with the second wave. Hence the similarity between the observed patterns of associations is suggestive of the crucial role perceived severity has on social contact behaviour. Our findings were echoed greatly by results from a study utilizing CoMix data from the United Kingdom (UK)^32^. This study found that individuals aged between 18 and 59 years who perceived high levels of seriousness if infected by the SARS-CoV-2 virus had lower mean number of contacts than those who perceived low levels of seriousness.

Several studies examined the role of risk perceptions on adoption of recommended preventive measures during the COVID-19 pandemic^7,12,15–22^. These studies have found that perceived severity was associated with the adoption of the protective behaviours, in line with the Health Belief Model. More specifically, people with higher perceived severity of the disease were found to be more likely to adopt the recommended precautionary measures. However, it is important to mention that the response variable of interest differed between studies. Whilst the response variable in our study was the number of social contacts, other studies considered indicators of avoidance of behaviour or adoption of the recommended measures as their outcome. Nonetheless, the results all point towards the critical role of perceived severity on individual’s response behaviour. Furthermore, the differences in the number of contacts for individuals with high perceived severity versus individuals with low or neutral levels of severity was around one contact in our study. The evaluation of the implications of such differences on the transmission dynamics of SARS-CoV-2 are a topic for future research. In addition, with respect to other response variables, the number of contacts can more easily and more consistently be included in mathematical models of infectious diseases^33^, making the analysis presented in this work crucial for future modelling endeavours of COVID-19.

In our study, the relationship between perceived susceptibility and the number of social contacts did not yield consistent relationships. These ambiguities may have resulted from a variety of factors including, but not limited to: firstly, there could be the presence of optimism bias, a phenomenon where individuals tend to underestimate their likelihood of experiencing a negative event or overestimate the likelihood of positive events^34^. In the context of the COVID-19 pandemic, this refers to individuals underestimating their perceived risk of getting infected. Several studies have indicated the presence of optimism bias during the COVID-19 pandemic^12,14,35^. Secondly, individuals having a higher number of social contacts might perceive themselves more likely to get infected as a result of their behaviour and vice-versa. Results from the aforementioned study in UK^32^ found that in general, participants who indicated to be likely to get infected by the SARS-CoV-2 virus had higher mean number of contacts than those who indicated to be unlikely to get the virus. And thirdly, this could be due to individuals’ perception on their inherent vulnerability to infection. Thus based on our results, the relationship between perceived susceptibility and social contact behaviour remains inconclusive and thus warrants more research.

Similarly, the relationship between perceived benefit to vulnerable and number of social contacts yielded inconsistent results. There were no significant differences in social contact behaviour between individuals who had high, neutral or low perceptions in terms of protecting the vulnerable individuals in the population. This could be due to either participants responding to the questionnaire item based on the frequency of contacts with vulnerable individuals within their close social circle or occupation (i.e, health care workers in elderly homes). In addition, it might be that participants who are vulnerable (mainly elderly people with underlying comorbidities) perceive no major benefit to other vulnerable individuals as they generally make fewer social contacts. As such, more research is required in this perspective as deliberate efforts in the realm of public health messaging and communication has emphasized on adhering to recommended measures to protect others^36^.

Perceived effectiveness of measures and perceived adherence to measures were both inversely associated with the number of contacts. Participants with high levels of perceived effectiveness of measures made lower number of contacts than those with low levels. Similarly, participants with high levels of perceived adherence to measures made fewer contacts than those low levels. However, the observed differences were generally small. According to the theory of Protection Motivation and Self-efficacy, persons’ belief in effectiveness of an intervention measure, and their confidence to adhere to the measure predicts the likelihood of engaging in the preventive behaviour^24^. Previous studies conducted under this theoretical framework—that explore the relationships between perceived effectiveness of measures and perceived adherence to measures with the recommended health behaviour—do not explicitly use the number of social contacts as a proxy of the recommended health behaviour. Instead, they use indicators of avoidance of behaviours or adoption of recommended measures as above-mentioned. However, our results are consistent with results from previous studies^4–6,15,25,26,37^ despite the outcome variables being slightly different. It is worth mentioning that the number of social contacts is a proxy of contact events responsible for disease transmission and is influenced by underlying determinants such as household size, day of the week (weekday versus weekend), age, among others as indicated in our study as well as in previous studies^27,38^. Thus, more studies utilizing the number of social contacts as a proxy of the adoption of recommended measures will be pertinent to shed more light on the influence of perceptions on contact behaviour, while controlling for possible confounders. Furthermore, data on perceived effectiveness of measures and perceived adherence to measures was only collected in the first 8 waves (i.e, the first wave of COVID-19 pandemic), and thus continued data collection on these contextual factors could be of great importance to gain additional insights in the observed relationships. It is worth mentioning that both the perceptions and number of social contacts changed over time with slight differences observed by age groups. Furthermore, the wave of data collection which coincided with changing regimes of intervention measures and also changing landscape of the pandemic, was an important factor in the interaction effects of the perception variables, further highlighting that perceptions and social contact behaviour were dynamic in time. This is consistent with results from 2 studies that found evolution of both perceptions and protective behaviours during the influenza A(H1N1)v2009 pandemic^9,10^, and a recent study from UK conducted during the COVID-19 pandemic^22^.

Our findings highlight the importance of aligning the public’s COVID-19 related perceptions with reality. That is, people who perceive COVID-19 to be more severe, will be more inclined to engage in preventive behaviours (here measured as the number of social contacts). Based on our results, we can suggest that public health communication and targeted messaging could yield more impact if tailored to messages emphasizing the severity of COVID-19. Thus, it is important to stress the severity of COVID-19—e.g in terms of excess mortality^39^ or long-term effects post COVID-19 infection^40^. Furthermore, we found significant interaction effects between age and both perceived severity and perceived susceptibility, hence age-adjusted campaigns with respect to disease severity and susceptibility are required to enhance social distancing measures. A collaborative multidisciplinary approach by scientists, policymakers and communication experts is pivotal to formulate an effective and contextualized strategy that could optimise the impact of public health messaging^41^.

Our study has several limitations. The associations between the perception variables and number of social contacts could have been affected by the level of stringency of the intervention measures that were being implemented. For example, during a lockdown, participants may not be able to contact people outside their household, even if they wanted to. However, this effect should be minimal as we controlled for the survey wave of data collection where different intervention measures were put in place. Information on COVID-19 vaccination was only partly available during the second survey. The percentage of the vaccinated individuals ranged from 0.5% in wave 12 to 14.8% in wave 19. Hence, due to the small sample of the vaccinated respondents, the vaccination status was not included in the analyses. However, a descriptive analysis (Supplementary Fig. S13) revealed no apparent differences in risk perceptions in the vaccinated individuals (before and after vaccination), and also in social contact behaviour between the vaccinated and not vaccinated (Supplementary Fig. S14). Although the panel of participants was representative by gender, age and region of residence in each survey wave, the voluntary opt-in of participants in each subsequent survey wave could be subject to self-selection bias where individuals more concerned about the pandemic in general would be more likely to participate. However, the participation rate was relatively high with 67.5% having participated in 3 or more waves in the first 8 survey waves and 63.19% in the subsequent 11 survey waves. Based on their importance in the context of social contact behaviour—we made sure the sampling design ensured representativeness in terms of age, gender and region of residence^27,38^. However, other potential factors such as race, urban/rural dwelling, income and education were not considered. Future studies of social contact patterns could take the latter factors into account to obtain an even more representative sample and to assess the impact of these factors on social contact patterns. In the process of model building for the different perception variables which entailed numerous hypothesis testing, our models could have missed potential significant interaction effects other than the ones we mainly focused on in our exploratory modeling. The latter were selected due to their epidemiological relevance in the SARS-CoV-2 pandemic. This could have marginally affected the significance of the terms in the final models in our analyses. While potentially having an impact on respondents’ risk perception, we did not collect information about their COVID-19 infection history. However, given the study was conducted in the early phases of the COVID-19 pandemic, where the percentages of the already infected in the population ranged between 0.04% and 0.62% in the first survey, and 4.05% and 7.72% in the second survey, the population-level effect is expected to be minimal. Our study findings could be subject to reverse causality since we assumed that perceptions precede the social contact behaviour, which might not necessarily be true. Our study could also suffer from social desirability bias, despite that anonymity of responses was assured. The results of our study apply to the Belgian population and caution is required when extrapolating these to other populations.

This study assessed the relationship between COVID-19 perceptions and social contact behaviour using two longitudinal surveys from a panel of individuals between April and August 2020, and November 2020 and April 2021 in Belgium. We found that individuals who perceived COVID-19 to be a serious illness for them made a significantly lower number of contacts as compared to those who had low or neutral perceptions. Similarly, individuals with high levels of perceived effectiveness and perceived adherence to measures made fewer contacts as compared to those with low levels. Given the importance of human behaviour in the transmission dynamics of SARS-CoV-2 virus, tailored communication strategies by public health officials about the severity of COVID-19 is crucial.

## Methods

### Ethics statement

Ethics approval for the study was given by the ethics committee of Antwerp University Hospital (reference number EC UZA 20/13/147). Participants aged 18 years and older opted to voluntarily participate in the study. In the second longitudinal survey, in addition, parents were invited to provide data on behalf of their child (a randomly selected child if a parent had several children). Informed consent was obtained from all subjects involved in the study. All methods were performed in accordance with the relevant guidelines and regulations.

### Survey methodology

**Figure 1:**
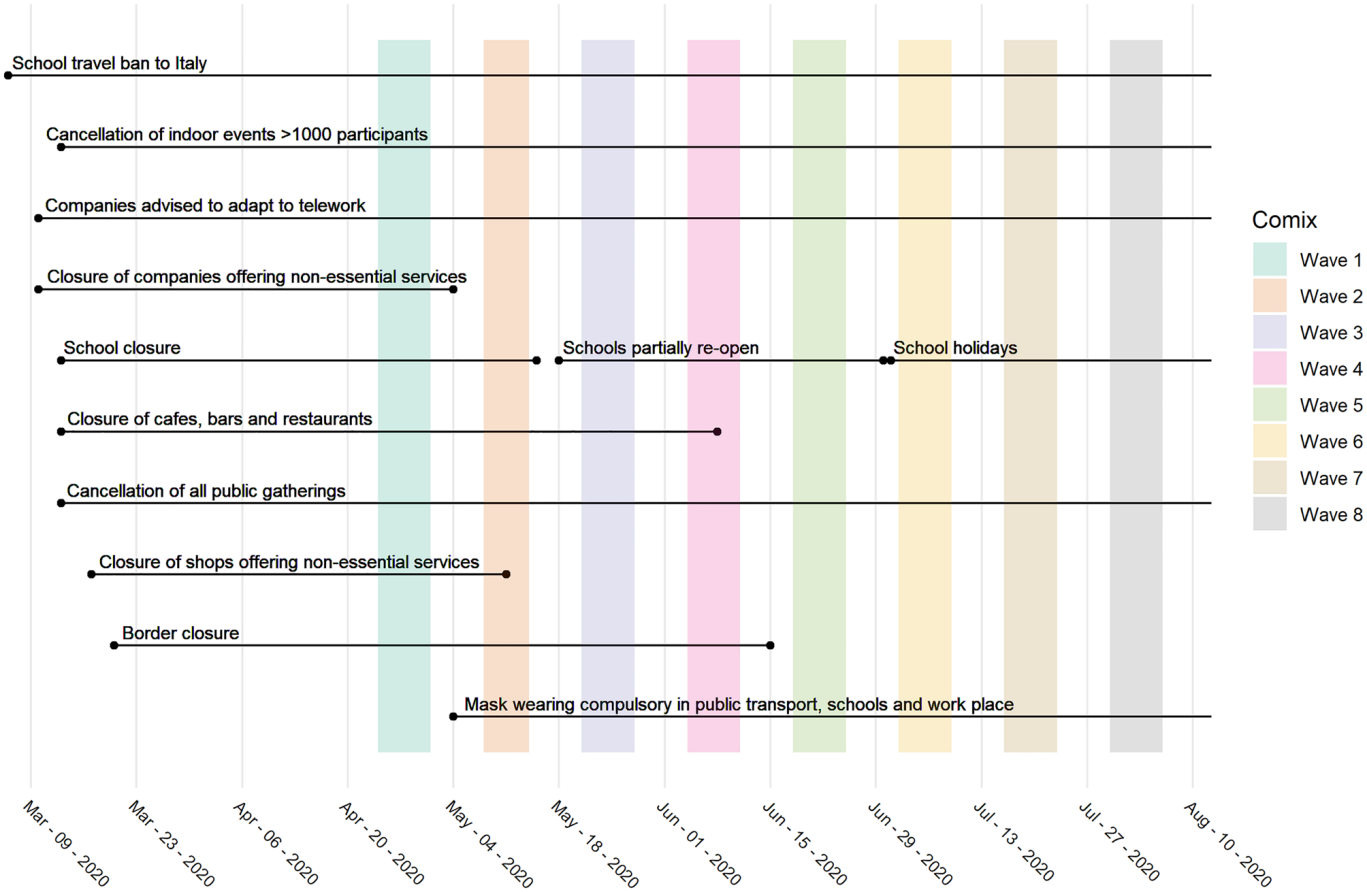
Calender of non-pharmaceutical interventions (NPIs) and CoMix waves for the first survey (waves 1–8). The Figure has been adapted from^30^.

The survey methodology and the sample characteristics for the first survey have been described by Coletti et al.^30^. Briefly, the first survey involved 8 waves of data collection in a representative panel of adults in terms of age, gender, and region of residence. More specifically, the panels of adults in the waves of data collection aimed for the same individuals filling out multiple surveys. Thus, a large proportion of responses were from the same participants. However, due to respondents’ drop-out in subsequent waves of data collection, the panel was topped up with additional individuals to match the target quota. For more information on the representativeness of the sample in comparison with the Belgian population, see Supplementary Table S1 that provides more details on the composition of the sample in each survey wave of data collection for the above-mentioned factors. These 8 waves coincided with different regimes of intervention measures that were implemented (Fig. 1). Although much of the survey methodology is outlined in^30^, below we highlight some factors that are deemed relevant for the current study. These include participants’ age, gender, information on socio-economic status, high-risk status, number of social contacts, perception related to risk of infection, perception towards the effectiveness of the imposed intervention measures and confidence to adherence to the intervention measures. The high-risk status corresponds to one or more of the following health conditions; chronic respiratory disease, chronic kidney disease, chronic heart disease, chronic neurological disease, chronic liver disease, immunosuppression, diabetes (all types), asplenia or dysfunction of the spleen, class III obesity, and pregnancy. The number of social contacts were measured between 5 am the day preceding the survey and 5 am of the survey day and represented all contacts that the participant made.

**Figure 2:**
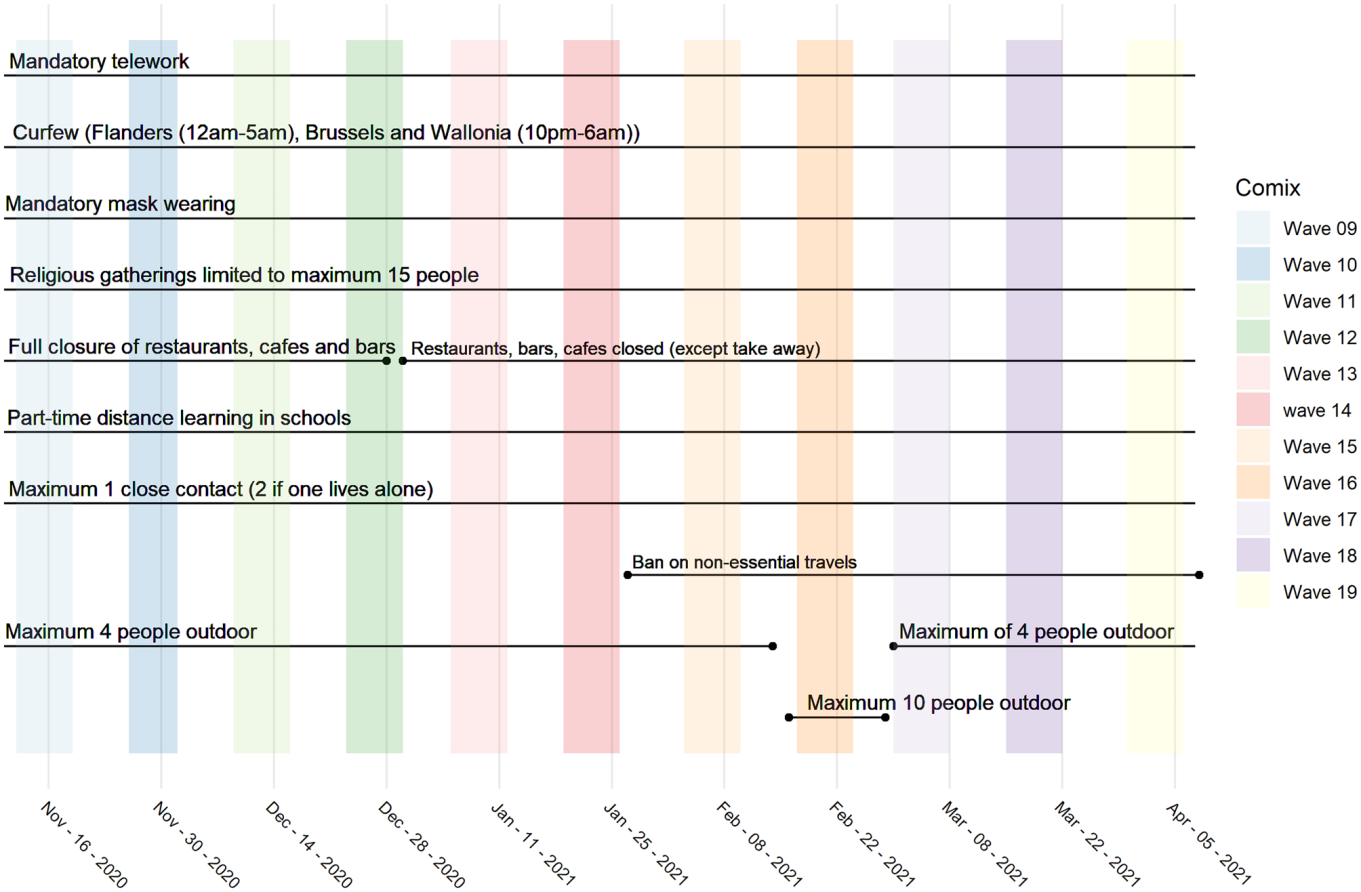
Calender of NPIs and CoMix waves for the second survey (waves 9–19).

**Figure 3:**
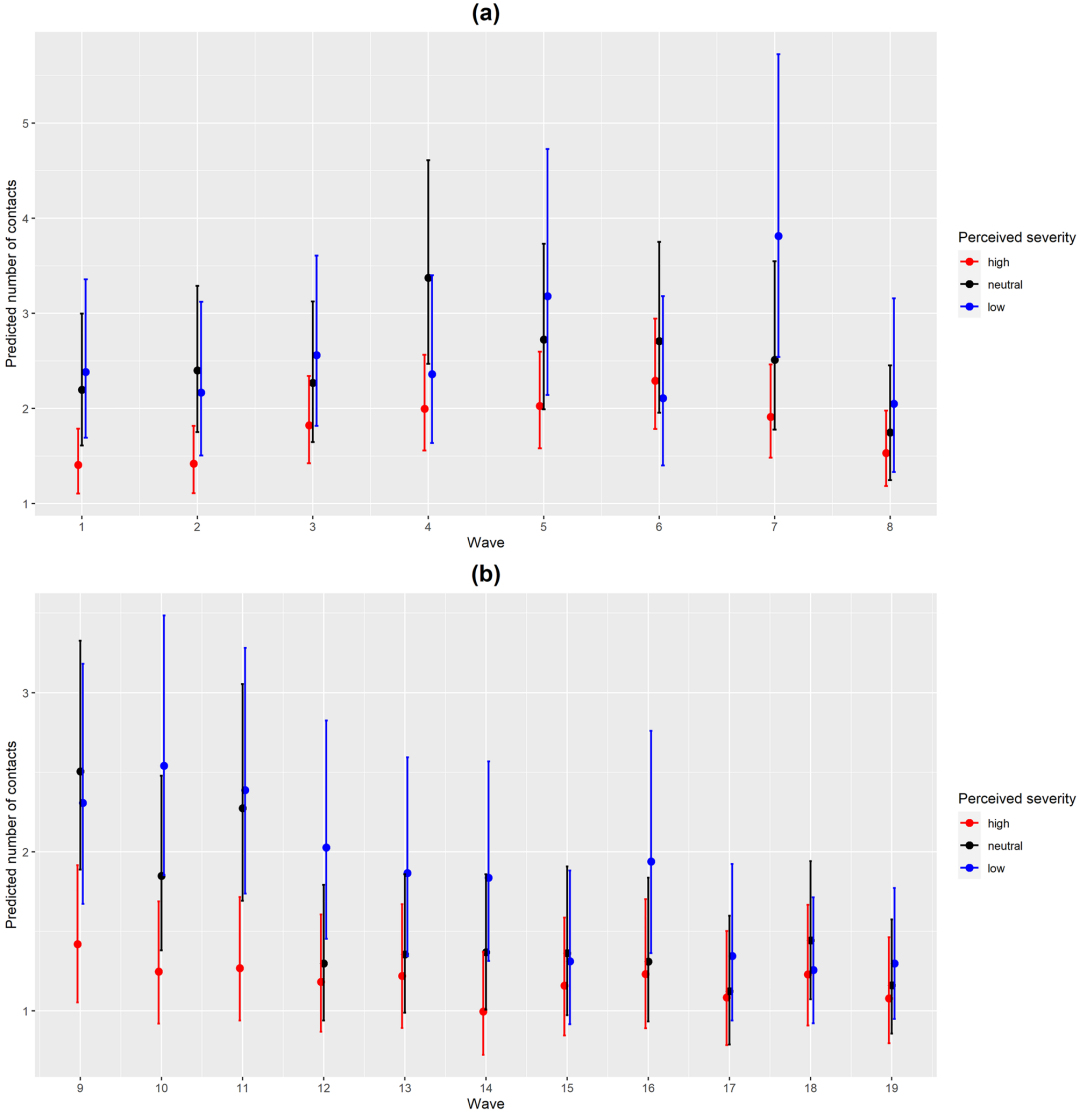
(**a**) Predicted number of contacts by perceived severity and wave with 95% CI for the first analysis from the perceived severity model. (**b**) Predicted number of contacts by perceived severity and wave with 95% CI for the second analysis from the perceived severity model.

**Figure 4:**
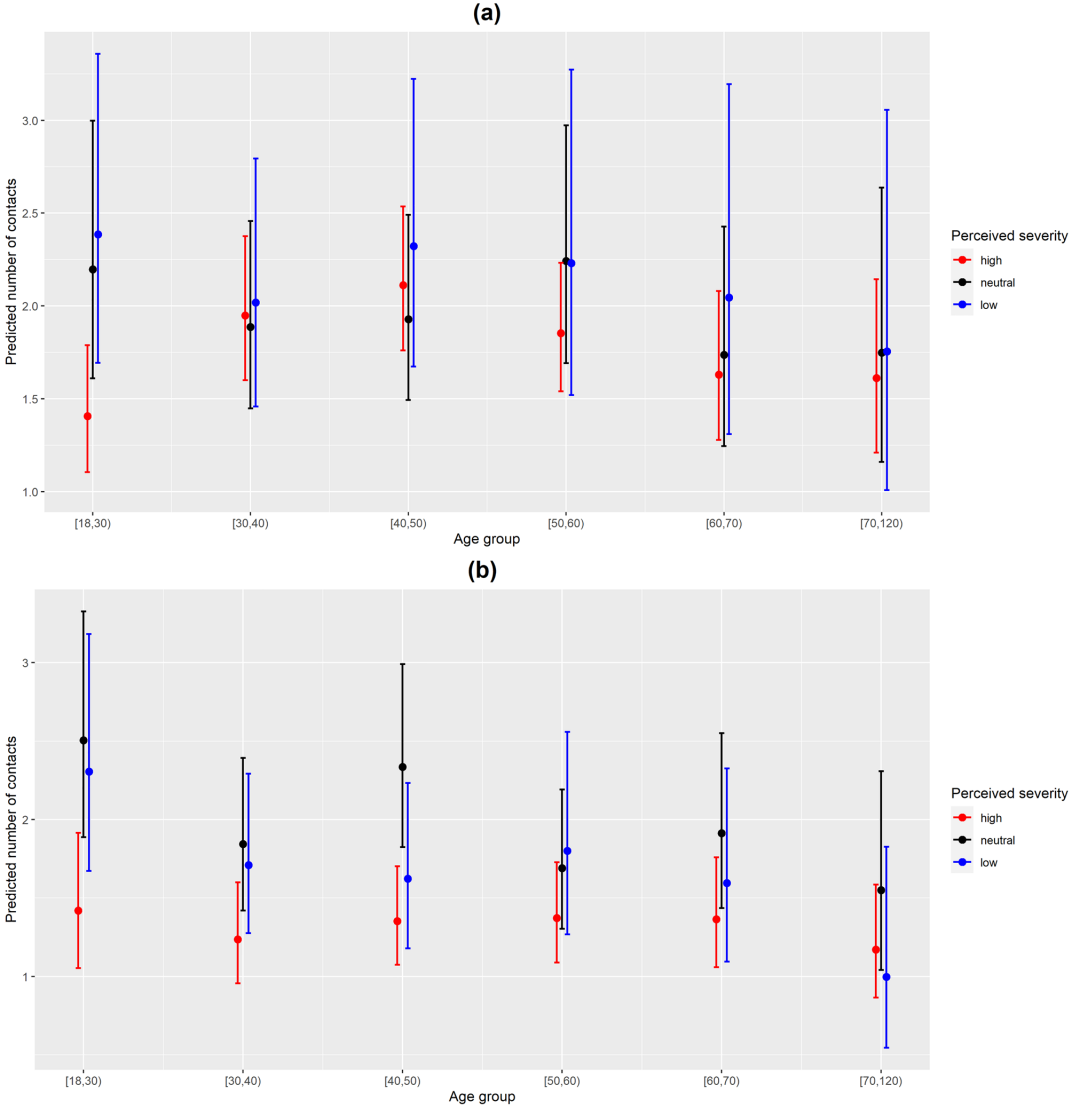
(**a**) Predicted number of contacts by perceived severity and age with 95% CI for the first analysis from the perceived severity model. (**b**) Predicted number of contacts by perceived severity and age with 95% CI for the second analysis from the perceived severity model.

The second survey involved 11 waves of data collection and corresponds largely to the second wave of the COVID-19 pandemic in Belgium (Fig. 2). The survey also ensured representativeness in terms of age, gender and region of residence. More information is contained in the Supplementary Table S2. The questionnaire used in the first survey was modified to collect data on behalf of children. The questionnaire items of the risk related perceptions remained the same in both surveys. However, questions related to perception on effectiveness of intervention measures and confidence to adhere to the measures were not collected in the second survey due to limitations on survey length. Similarly during data collection in subsequent waves, as the original panel loses some participants in specific waves, the cohort is replenished to fulfil sample size requirements. We utilize these two unique longitudinal surveys collected during the first and second wave of COVID-19 in Belgium to explore how perceptions relate with changes in social contacts behaviour. Thus our study provides a good basis to understand the link between perceptions and number of social contacts during the current pandemic.

**Table 1:** Correspondence between analysis variables—risk perception, perception on effectiveness of intervention measures and confidence to adhere to interventions—and questionnaire items.

Variables	Questionnaire items
**Risk Perception**$^{{\text {a,b}}}$ Strongly agree Tend to agree Neither agree nor disagree Tend to disagree Strongly disagree Don’t know	**To what extent do you agree or disagree with the following statements ...** Coronavirus would be serious illness for me (**Perceived severity**) I am likely to catch coronavirus (**Perceived susceptibility**) If I don’t follow the government’s advice, I might spread coronavirus to someone who is vulnerable (Perceived benefit to vulnerable)
**Perception on effectiveness**$^{{\text {a}}}$ **of intervention measures** Very effective Fairly effective Not very effective Not at all effective Don’t know	**How effective, if at all, do you think ... is at slowing the spread of coronavirus?** Reducing the number of people you meet Staying at home for 7 days if you have a mild symptom such as a mild cough Staying at home for 7 days if you have more severe symptoms such as a severe cough or a high temperature Avoiding crowded places Stay at home for 14 days if anyone other than yourself in your household has mild symptom such as a mild cough Stay at home for 14 days if anyone other than yourself in your household has severe symptoms such as a cough or a high temperature School closures Banning the use of public transport Closing bars, restaurants, cinemas etc.
**Perception on confidence**$^{{\text {a}}}$ **to adhere to measures** Very confident Fairly confident Not very confident Not at all confident Don’t know	**How confident are you, if at all, that if you wanted to you could ... ?** Reduce the number of people you meet Stay at home for 7 days if you have a mild symptom such as a mild cough Stay at home for 7 days if you have more severe symptoms such as a severe cough or a high temperature Avoid crowded places Stay at home for 14 days if anyone other than yourself in your household has mild symptom such as a mild cough Stay at home for 14 days if anyone other than yourself in your household has severe symptoms such as a cough or a high temperature Not use the public transport

$^{{\text {a}}}$Information collected during the first survey (8 waves). $^{{\text {b}}}$Information collected during the second survey (11 waves).

To characterize the risk perception, participants were asked to indicate their level of agreement or disagreement on 3 items. See Table 1 for full item wordings. To characterize the perception on the effectiveness of intervention measures, participants had to indicate their perception on the level of effectiveness on each of 9 social and behavioral intervention measures. The responses were categorised into 5 categories; very effective, fairly effective, not very effective, not at all effective, and don’t know (Table 1). In terms of the confidence to adhere to the imposed measures, participants were provided with 7 items corresponding to the intervention measures and asked to indicate their level of confidence, that they could adhere to these measures if they wanted to. The responses were grouped into five categories; very confident, fairly confident, not very confident, not at all confident, and don’t know (Table 1).

### Data pre-processing

We coded the items corresponding to risk perceptions on 5-point Likert scales. Items corresponding to the perception on effectiveness of intervention measures and confidence to adhere were converted into 4-point Likert scales (Table 1). Since participants were also given an option *don’t know* in the list of responses, in the process of coding these items into Likert-scales, this option was treated as a missing value. This was solely to enhance exploratory analysis for the internal consistency of the items and also for the computation of the mean changes of the perceptions over time. Hence, in the statistical analyses, it was included as a response level as explained in the next paragraph.

We used Cronbach’s alpha as the reliability measure of internal consistency of the Likert-scales^42^. We considered a threshold of 0.80 of the Cronbach’s alpha reliability coefficient to group the items^42^. Items corresponding to the risk perceptions had Cronbach’s alpha of 0.5 (95% confidence interval (CI) 0.48–0.52) and 0.52 (95% CI 0.50–0.53), in the first and the second survey, respectively. Thus, the three items could not be combined into one variable representing the underlying risk perception construct. Therefore, we considered the risk perception questions as three separate constructs (i.e, perceived severity for the item *“coronavirus would be a serious illness for me”*, perceived susceptibility for the item *“I am likely to catch coronavirus”*, and perceived benefit to the vulnerable for the item *“If I don’t follow the government’s advice, I might spread the coronavirus to someone who is vulnerable”*, see Table 1). Motivated by exploratory analyses, we categorised the 6 response levels into three response levels: high perception (‘strongly agree’ and ‘tend to agree’), low perception (‘tend to disagree’ and ‘strongly disagree’), and neutral (‘neither agree nor disagree’ and ‘don’t know’).

Cronbach’s alpha for the 9 items of perceived effectiveness of measures was 0.86 (95% CI 0.85–0.87) and for the 7 items of perceived adherence to measures was 0.87 (95% CI 0.86–0.88), thus we obtained composite scores for the perceived effectiveness and perceived adherence to measures. More information on the survey items is contained in Table 1. In the remainder of this paper, we use the terms *perceived severity*, *perceived susceptibility* and *perceived benefit to vulnerable* for the risk related perceptions. While *perceived effectiveness of measures*, and *perceived adherence to measures* will be used for the perceptions on effectiveness of measures and confidence to adhere to the measures.

## Statistical analyses

We used a generalized linear mixed effects model (GLMM) to model the number of contacts in the two surveys^43^. We used random effects in the model to incorporate the correlations among observations from the same participant. A negative binomial distribution allowing for overdispersion was used to define the error distribution, while we applied zero-inflation to deal with excess zeroes in the number of social contacts. The model adjusted for the survey wave (time of data collection as categorical variable), day of the week (weekday versus weekend), the participant’s household size, gender, age, face mask wearing and high risk status to control for possible confounding. The model was fitted using maximum likelihood estimation. We used R version 4.0.3 and the glmmTMB package (version 1.0.2.1)^44^ for all statistical analyses.

Model building was performed for each individual perception variable. This was informed by preliminary exploratory analyses which identified significant interaction effects between the different perception variables and several control variables. Hence in total, we had 5 models for the first survey where each model represented an individual perception variable (i.e. *perceived severity*, *perceived susceptibility*, *perceived benefit to vulnerable*, *perceived effectiveness of measures*, and *perceived adherence to measures*). In the analysis of the second survey, we had 3 models where each corresponded to an individual risk-related perception (i.e. *perceived severity*, *perceived susceptibility*, *perceived benefit to vulnerable*) since these waves did not query for perception of effectiveness and confidence to adherence to measures. The significance of the variables in the models was assessed through Type III Wald tests and a significance level of 5% was considered. For convenience and ease of reporting results, the analyses involving data from the first survey (8 waves) will be termed as **first analysis**, whilst the one involving the second survey (11 waves) will be termed as **second analysis** in the rest of the work.

## Supplementary Information

Supplementary Information.

## Data Availability

The datasets utilized in this study will be made publicly available through the zenodo platform and any other additional information will be provided by the authors upon a reasonable request.
