# Supplementary material for: The influence of risk perceptions on close contact frequency during the SARS-CoV-2 pandemic
Source: Sci Rep. 2022 Mar 25;12:5192. doi: 10.1038/s41598-022-09037-8 (PMC8951651; doi:10.1038/s41598-022-09037-8)
Supplement: Supplementary file 1 — Supplementary Information. [file 41598_2022_9037_MOESM1_ESM.pdf]

The influence of risk perceptions on close contact frequency during the SARS-CoV-2 pandemic

James Wambua[1*], Lisa Hermans[1], Pietro Coletti[1], Frederik Verelst[2], Lander Willem[2], Christopher I Jarvis[3], Amy Gimma[3], Kerry LM Wong[3], Adrien Lajot[4], Stefaan Demarest[4], W. John Edmunds[3], Christel Faes[1], Philippe Beutels[2,5], Niel Hens[1,2]

[1]UHasselt, Data Science Institute, I-BioStat, Hasselt, 3500, Belgium.

[2]University of Antwerp, Centre for Health Economic Research and Modelling Infectious Diseases, Vaccine & Infectious Disease Institute, Antwerp, 2610, Belgium.

[3]Centre for Mathematical Modelling of Infectious Diseases, Department of Infectious Disease Epidemiology, London School of Hygiene and Tropical Medicine, Keppel Street, WC1E 7HT London, UK.

[4]Department of Epidemiology and public health, Sciensano, Brussels, Belgium.

[5]The University of New South Wales, School of Public Health and Community Medicine, Sydney, NSW 2033, Australia.

Supplementary Information

(a)

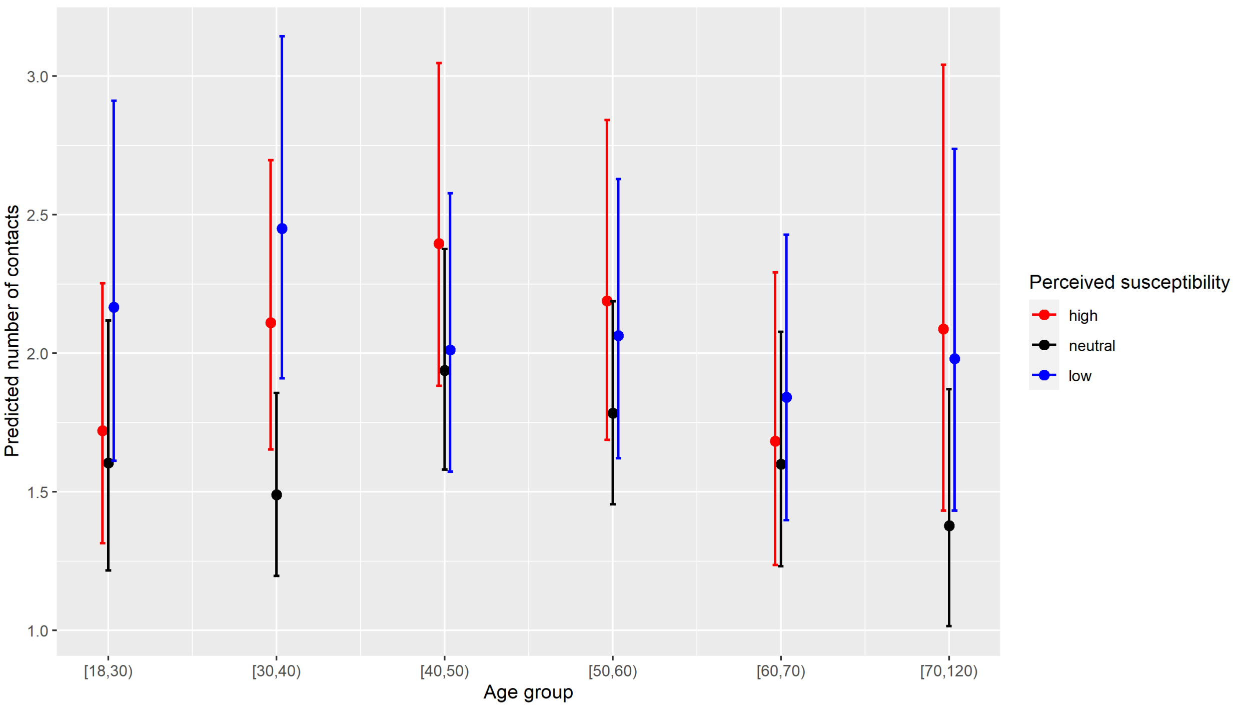

(b)

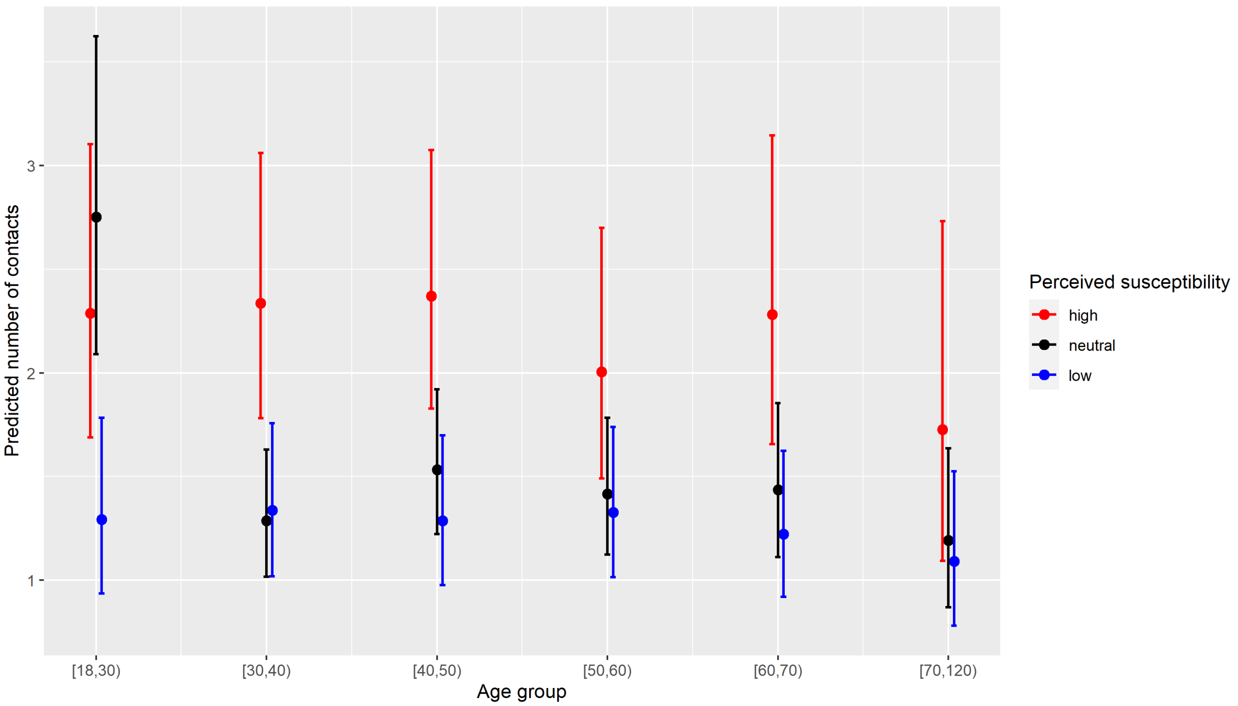

Figure S1: **(a):** Predicted number of contacts by perceived susceptibility and age group with 95% confidence interval (CI) for the first analysis from the perceived susceptibility model. **(b):** Predicted number of contacts by perceived susceptibility and age group with 95% CI for the second analysis from the perceived susceptibility model.

(a)

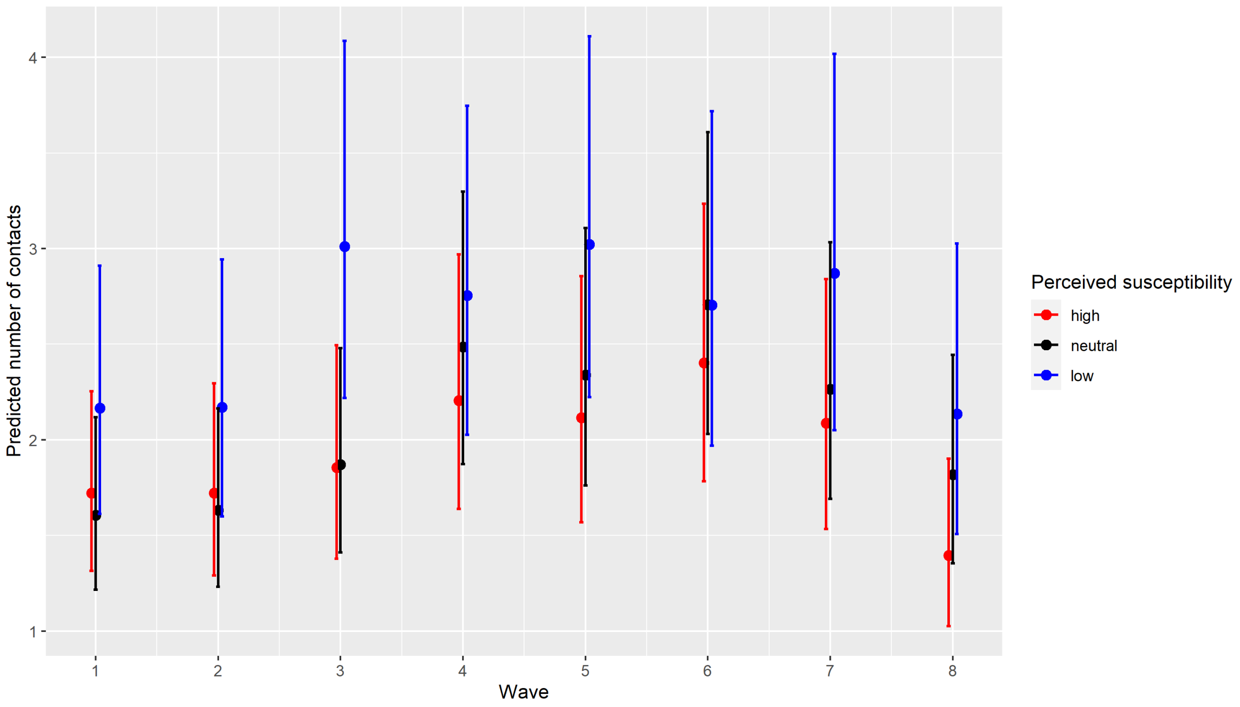

(b)

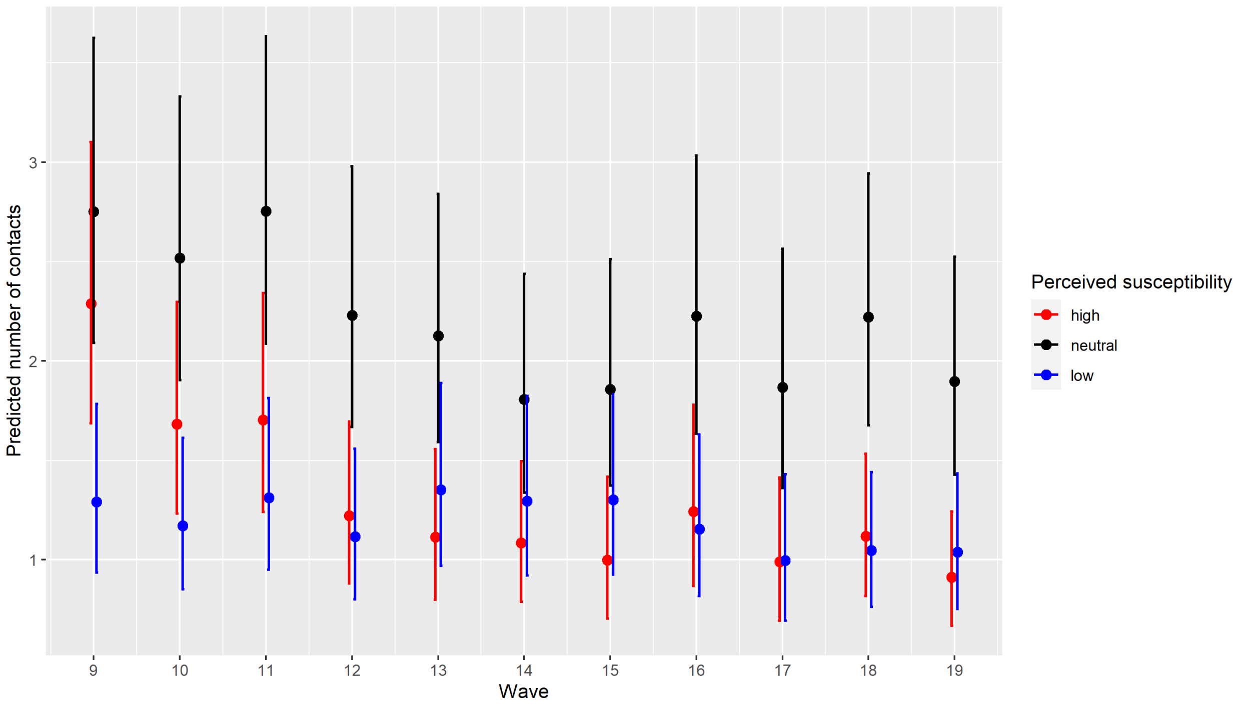

Figure S2: **(a):** Predicted number of contacts by perceived susceptibility and survey wave of data collection with 95% CI for the first analysis from the perceived susceptibility model. **(b):** Predicted number of contacts by perceived susceptibility and survey wave of data collection with 95% CI for the second analysis from the perceived susceptibility model.

(a)

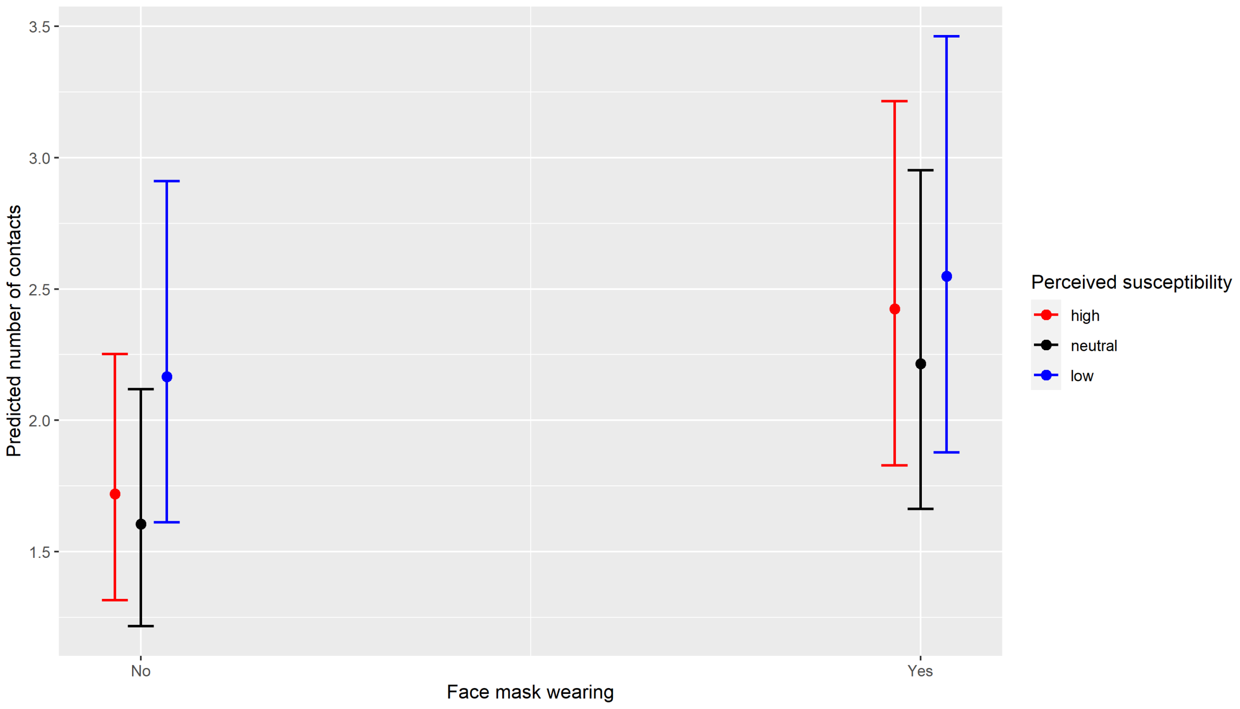

(b)

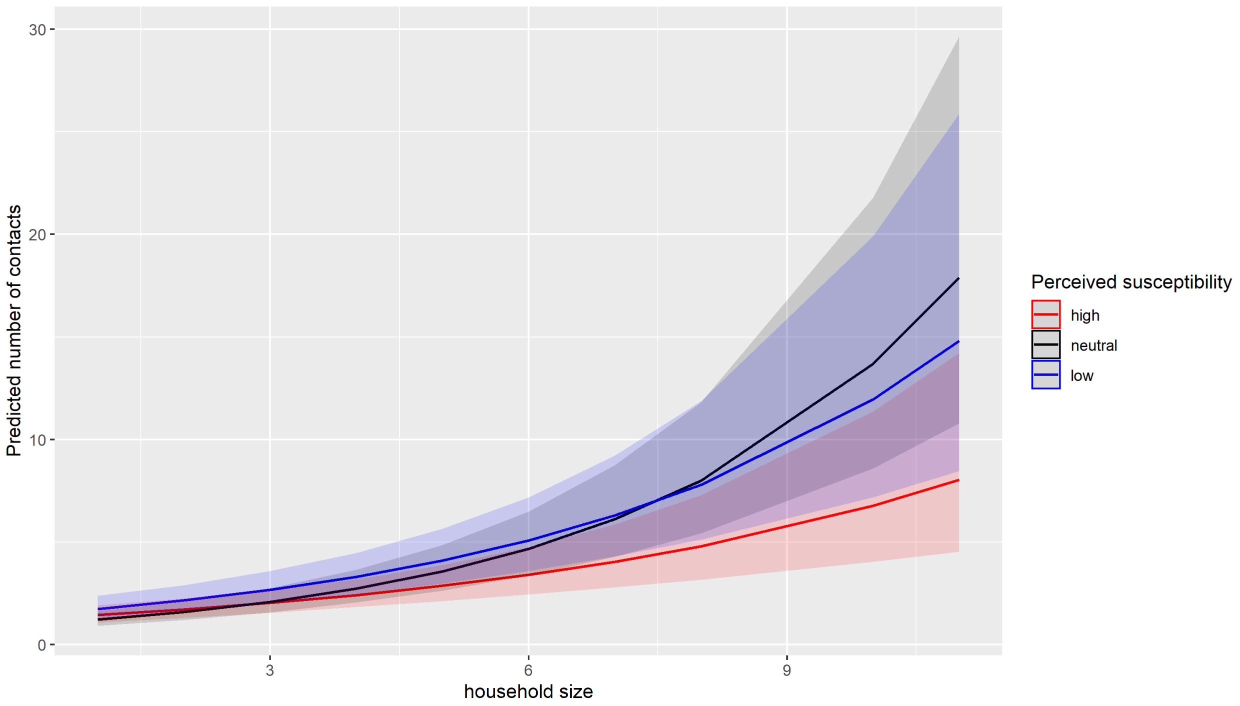

Figure S3: **(a):** Predicted number of contacts by perceived susceptibility and face mask wearing with 95% CI for the first analysis from the perceived susceptibility model. **(b):** Predicted number of contacts by perceived susceptibility and household size with 95% CI for the first analysis from the perceived susceptibility model.

(a)

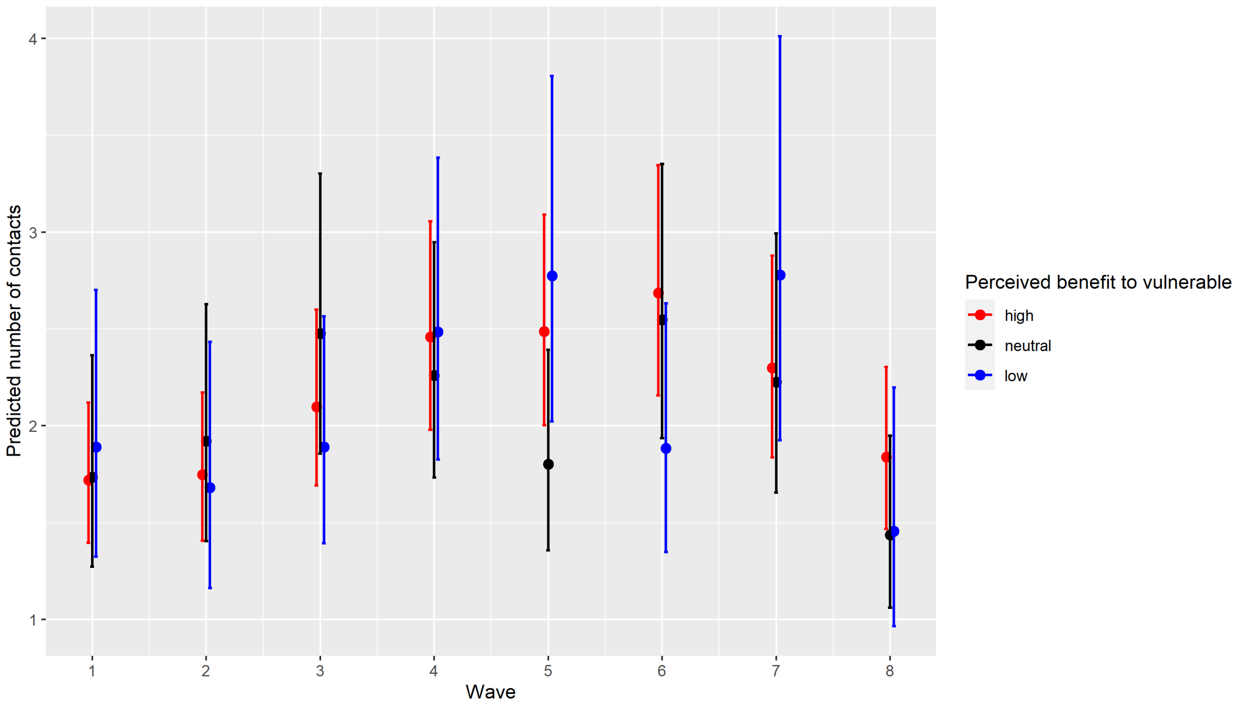

(b)

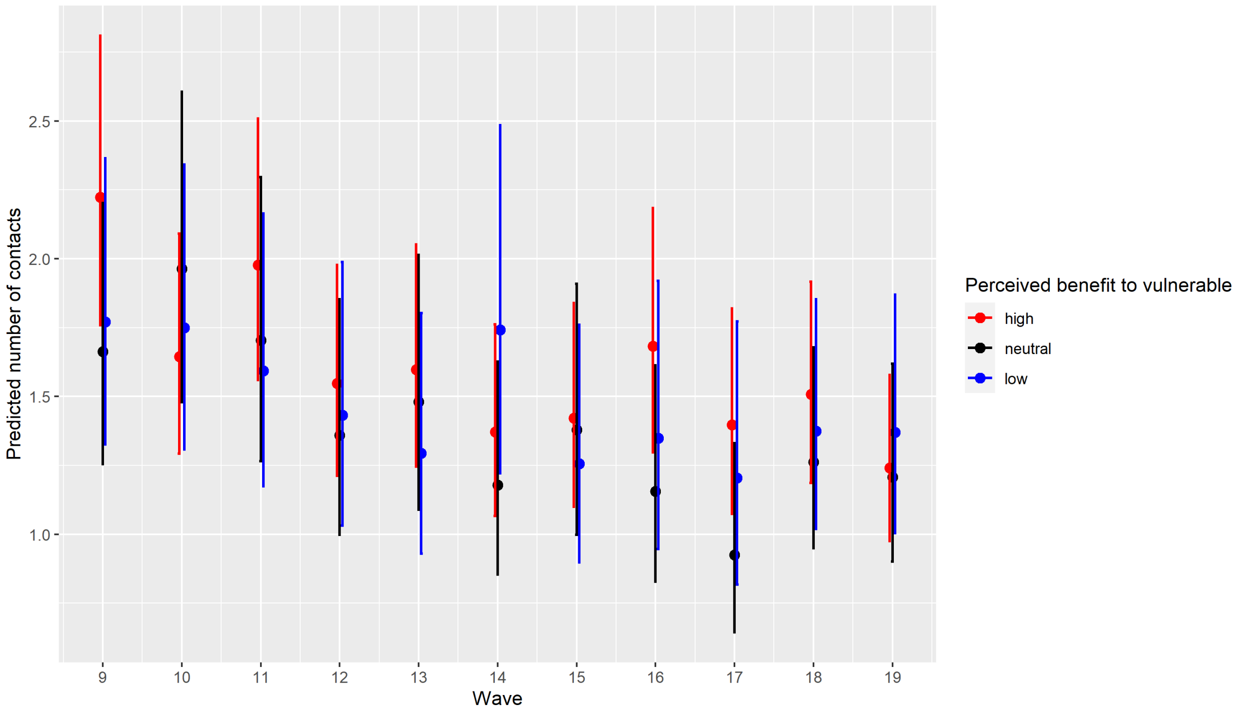

Figure S4: **(a):** Predicted number of contacts by perceived benefit to vulnerable and survey wave of data collection with 95% CI for the first analysis from the perceived benefit to vulnerable model. **(b):** Predicted number of contacts by perceived benefit to vulnerable and survey wave of data collection with 95% CI for the second analysis from the perceived benefit to vulnerable model.

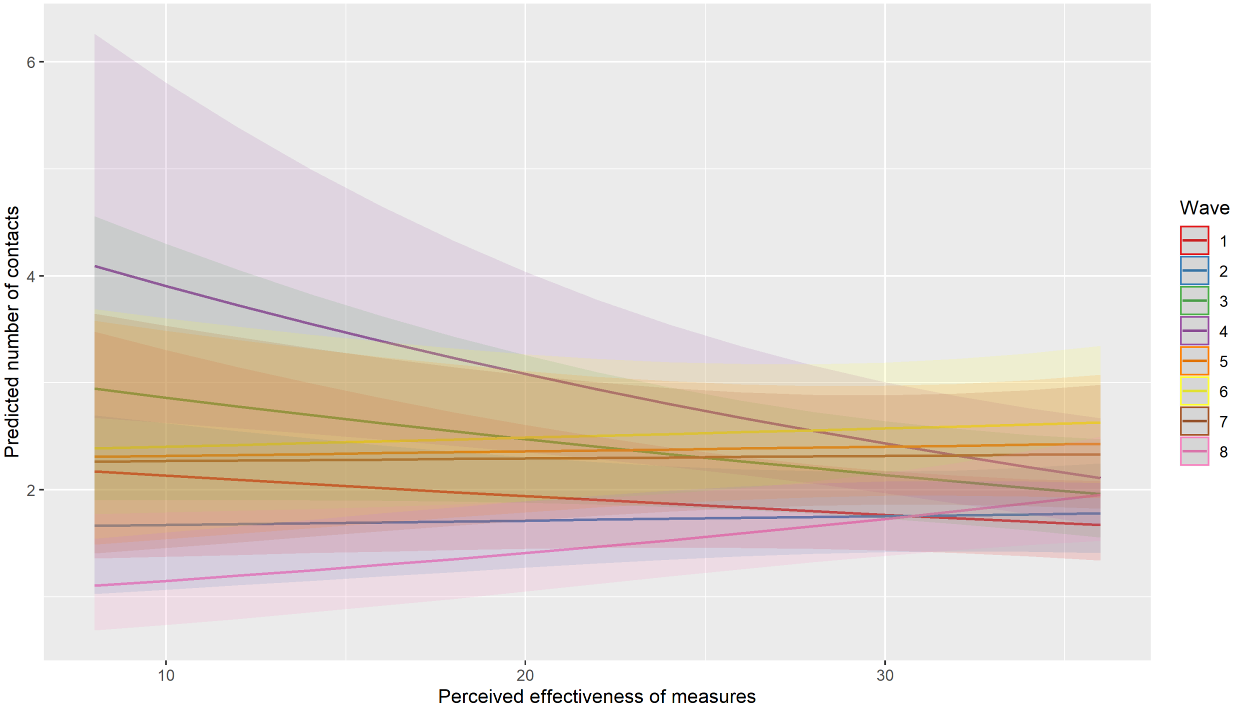

Figure S5: Predicted number of contacts by perceived effectiveness of measures and survey wave of data collection with 95% CI for the first analysis from the perceived effectiveness model.

	Population (1 Jan 2020)	Wave 1 (N=1542)	Wave 2 (N=1277)	Wave 3 (N=1142)	Wave 4 (N=951)	Wave 5 (N=924)	Wave 6 (N=902)	Wave 7 (N=760)	Wave 8 (N=833)
Region									
Brussels region	10.6%	110 (7.1%)	81 (6.3%)	75 (6.6%)	60 (6.3%)	57 (6.2%)	56 (6.2%)	48 (6.4%)	52 (6.3%)
Flanders	57.7%	961 (62.3%)	825 (64.6%)	732 (64.1%)	590 (62.0%)	572 (61.9%)	561 (62.2%)	496 (65.2%)	525 (63.0%)
Wallonia	31.8%	471 (30.5%)	371 (29.1%)	335 (29.3%)	301 (31.7%)	295 (31.9%)	285 (31.6%)	216 (28.4%)	256 (30.7%)
Age group									
0-9	11.1%	-	-	-	-	-	-	-	-
10-19	11.3%	35 (2.3%)	20 (1.6%)	11 (0.96%)	8 (0.84%)	10 (1.1%)	8 (0.89%)	4 (0.5%)	4 (0.5%)
20-29	12.3%	229 (14.9%)	156 (12.2%)	123 (10.8%)	89 (9.4%)	95 (10.3%)	84 (9.3%)	66 (8.7%)	78 (9.4%)
30-39	13.0%	222 (14.4%)	183 (14.3%)	157 (13.7%)	124 (13.0%)	121 (13.1%)	124 (13.7%)	82 (10.8%)	102 (12.2%)
40-49	13.1%	280 (18.2%)	233 (18.2%)	209 (18.3%)	156 (16.4%)	152 (16.5%)	145 (16.1%)	129 (17.0%)	138 (16.6%)
50-59	13.8%	310 (20.1%)	273 (21.4%)	253 (22.2%)	221 (23.2%)	198 (21.4%)	200 (22.2%)	177 (23.3%)	195 (23.4%)
60-69	11.7%	318 (20.6%)	286 (22.4%)	271 (23.7%)	243 (25.6%)	237 (25.6%)	231 (25.6%)	202 (26.6%)	207 (24.8%)
70+	13.7%	148 (9.6%)	126 (9.9%)	118 (10.3%)	110 (11.6%)	111 (12.0%)	110 (12.2%)	100 (13.1%)	109 (13.1%)
Gender									
Males	49.3%	732 (47.5%)	604 (47.3%)	560 (49.0%)	484 (50.9%)	463 (50.1%)	445 (49.3%)	386 (50.8%)	429 (51.5%)
Females	50.7%	810 (52.5%)	671 (52.5%)	581 (50.9%)	467 (49.1%)	460 (49.8%)	456 (50.6%)	374 (49.2%)	399 (47.9%)
NAs	-	-	2 (0.16%)	1 (0.09%)	-	1 (0.11%)	1 (0.11%)	-	5 (0.6%)

Table S1: Summary of sample characteristics for the first survey (waves 1-8). The table has been adapted from Table 1 of Coletti et al 2020.

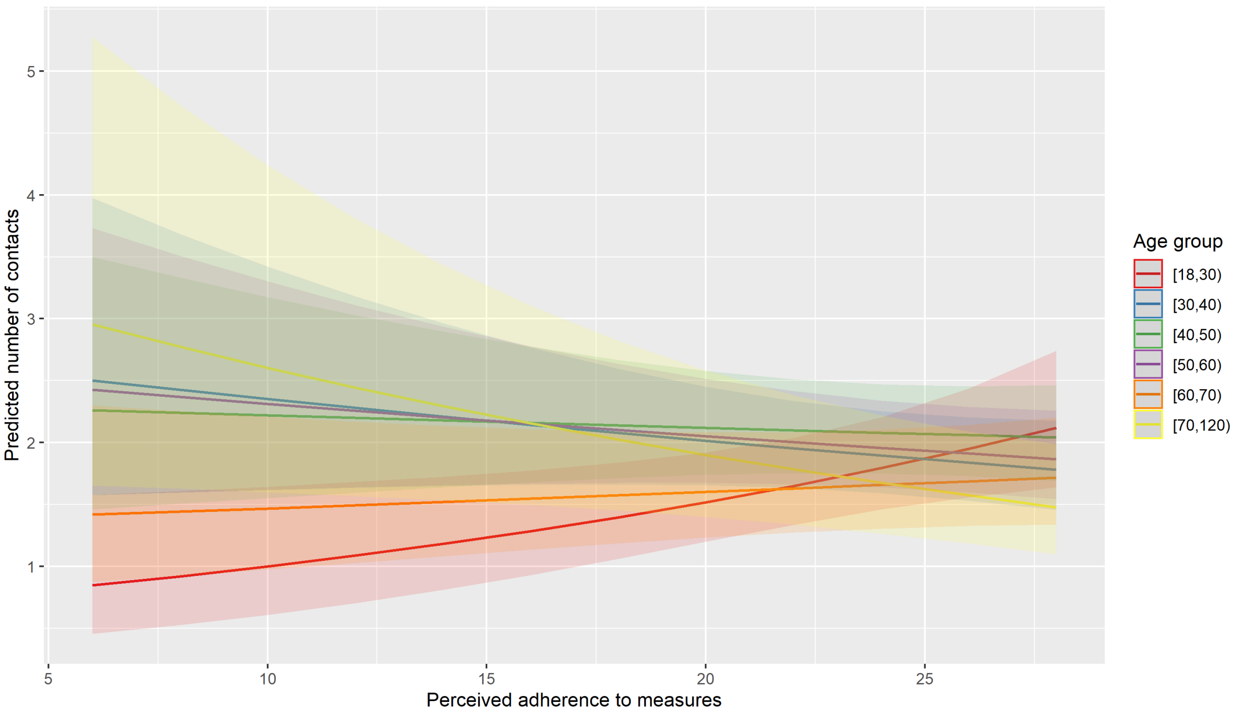

Figure S6: Predicted number of contacts by perceived adherence to measures and age with 95% CI for the first analysis from the perceived adherence model.

	Population (1 Jan)	Wave 9 (1500)	Wave 10 (1340)	Wave 11 (1209)	Wave 12 (1087)	Wave 13 (983)	Wave 14 (883)	Wave 15 (799)	Wave 16 (719)	Wave 17 (645)	Wave 18 (1497)	Wave 19 (1383)
Region												
Brussels	10.6% 10.6%	123 (8.2%)	114 (8.5%)	91 (7.5%)	76 (7.0%)	63 (6.4%)	64 (7.2%)	31 (3.8%)	25 (3.5%)	43 (6.7%)	115 (7.7%)	104 (7.5%)
Flanders	57.7%	894 (59.6%)	761 (56.7%)	718 (59.3%)	687 (63.2%)	611 (62.2%)	580 (65.7%)	649 (81.2%)	598 (83.1%)	423 (65.5%)	892 (59.6%)	856 (61.9%)
Wallonia	31.8%	483 (32.2%)	465 (34.7%)	400 (33.1%)	324 (29.8%)	309 (31.4%)	239 (27.1%)	119 (14.9%)	96 (13.3%)	179 (27.8)	490 (32.7%)	423 (30.6%)
Age												
0-18	11.1%	300 (20.0%)	263 (19.6%)	242 (20.0%)	220 (20.2%)	197 (20.0%)	178 (20.1%)	160 (20.0%)	144 (20.0%)	130 (20.1%)	299 (20.0%)	278 (20.2%)
18-29	14.6%	228 (15.2%)	180 (13.4%)	137 (11.3%)	126 (11.6%)	137 (13.9%)	137 (15.5%)	100 (12.5%)	84 (11.7%)	83 (12.8%)	221 (14.8%)	187 (13.5%)
30-39	13.0%	174 (11.6%)	125 (9.3%)	125 (10.3%)	116 (10.7%)	113 (11.5%)	121 (13.7)	81 (10.1%)	73 (10.2%)	76 (11.8%)	188 (12.6%)	150 (10.8%)
40-49	13.1%	195 (13.0%)	156 (11.6%)	173 (14.3%)	155 (14.3%)	136 (13.8%)	156 (17.7%)	114 (14.3%)	100 (13.9%)	87 (13.5%)	191 (12.8%)	175 (12.7%)
50-59	13.8%	191 (12.7%)	215 (16.0%)	176 (14.6%)	160 (14.7%)	137 (13.9%)	118 (13.4%)	121 (15.1%)	138 (19.2%)	89 (13.8%)	201 (13.4%)	201 (14.5%)
60-69	11.7%	285 (19.0%)	276 (20.6%)	249 (20.6%)	220 (20.2%)	184 (18.7%)	133 (15.1%)	145 (18.1%)	135 (18.8%)	156 (24.2%)	267 (17.8%)	258 (18.6%)
70+	13.7%	127 (8.5%)	125 (9.3%)	107 (8.9%)	90 (8.3%)	79 (8.0%)	40 (4.5%)	78 (9.7%)	45 (6.3%)	24 (3.7%)	130 (8.7%)	133 (9.6%)
Gender												
Males	49.3%	602 (50.2%)	534 (49.6%)	492 (50.8%)	537 (49.4%)	457 (46.5%)	408 (46.2%)	387 (48.4%)	355 (49.4%)	315 (48.8%)	713 (47.6%)	660 (47.7%)
Females	50.7%	597 (49.8%)	541 (50.2%)	475 (49.1%)	548 (50.4%)	525 (53.4%)	473 (53.6%)	411 (51.4%)	363 (50.4%)	330 (51.1%)	780 (52.2%)	719 (52.0%)
NAs	-	1 (0.08%)	1 (0.09%)	- -	1 (0.09%)	1 (0.1%)	2 (0.2%)	1 (0.1%)	1 (0.1%)	-	3 (0.2%)	3 (0.2%)

Table S2: Summary of sample characteristics for the second survey. The percentages are rounded to one decimal place and thus might not add up to 100%. The composition of gender from wave 9 to wave 11 is only considered for the adults.

(a)

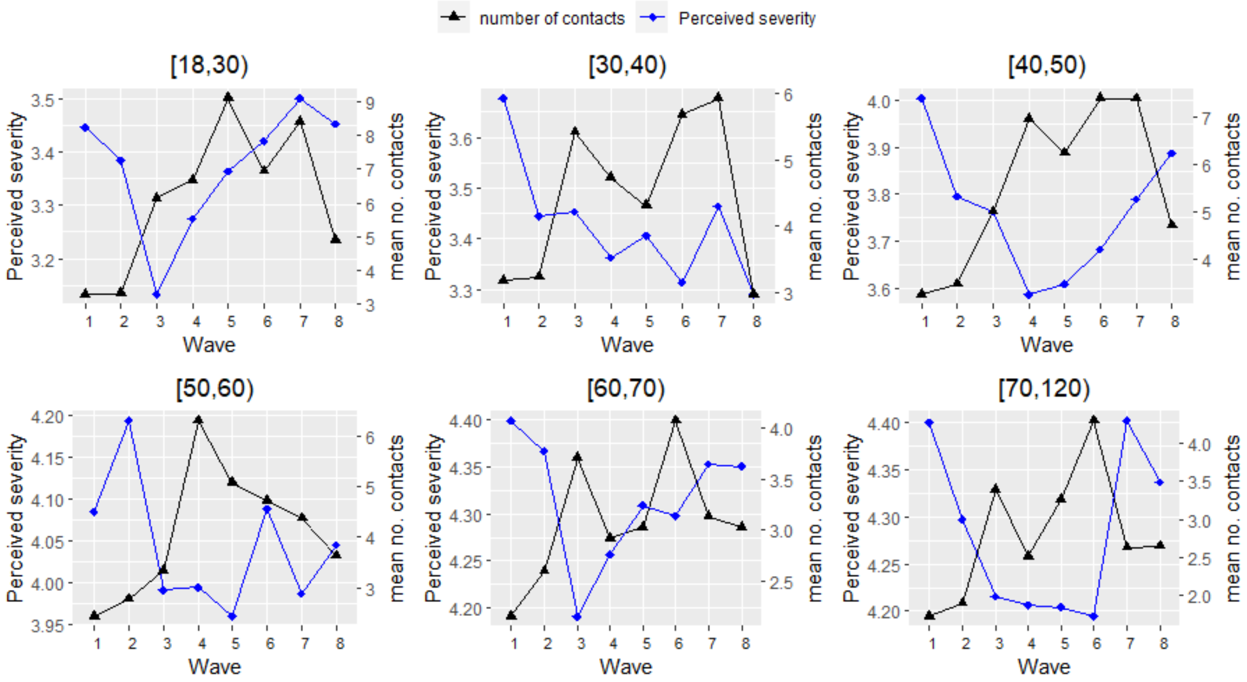

(b)

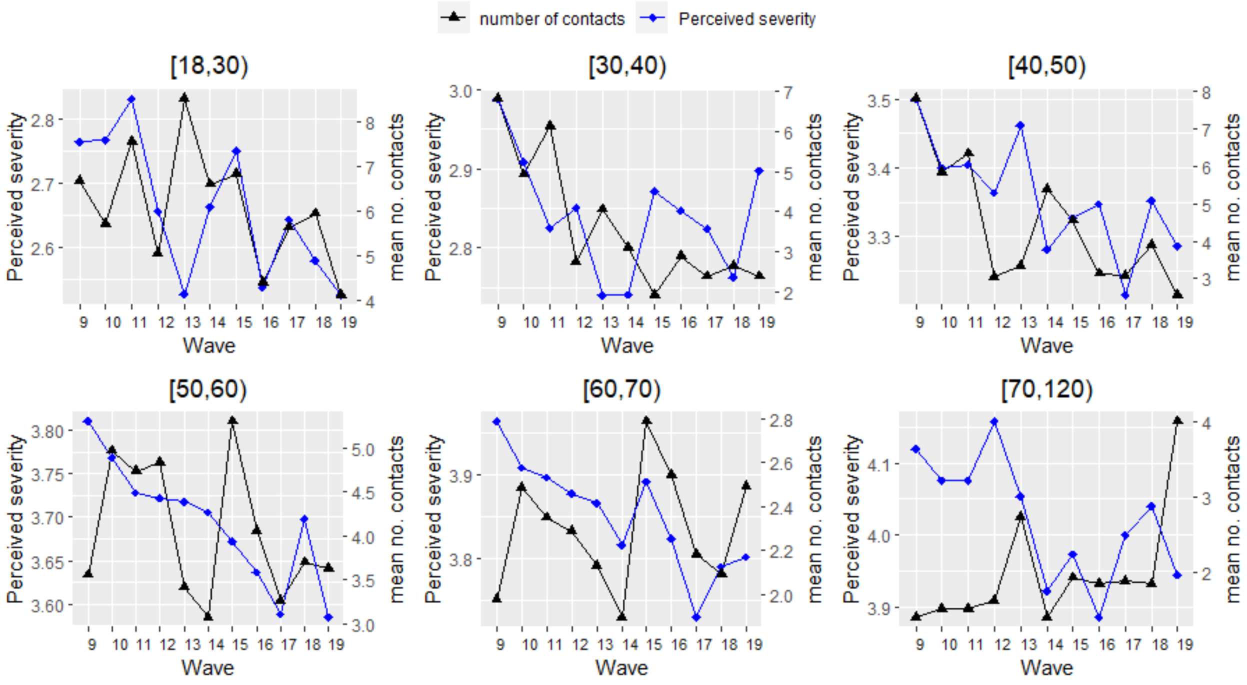

Figure S7: **(a):** Dynamics of perceived severity and the number of social contacts in the first analysis. **(b):** Dynamics of perceived severity and the number of social contacts in the second analysis. The lines show the mean score of the perceived severity and average number of social contacts.

(a)

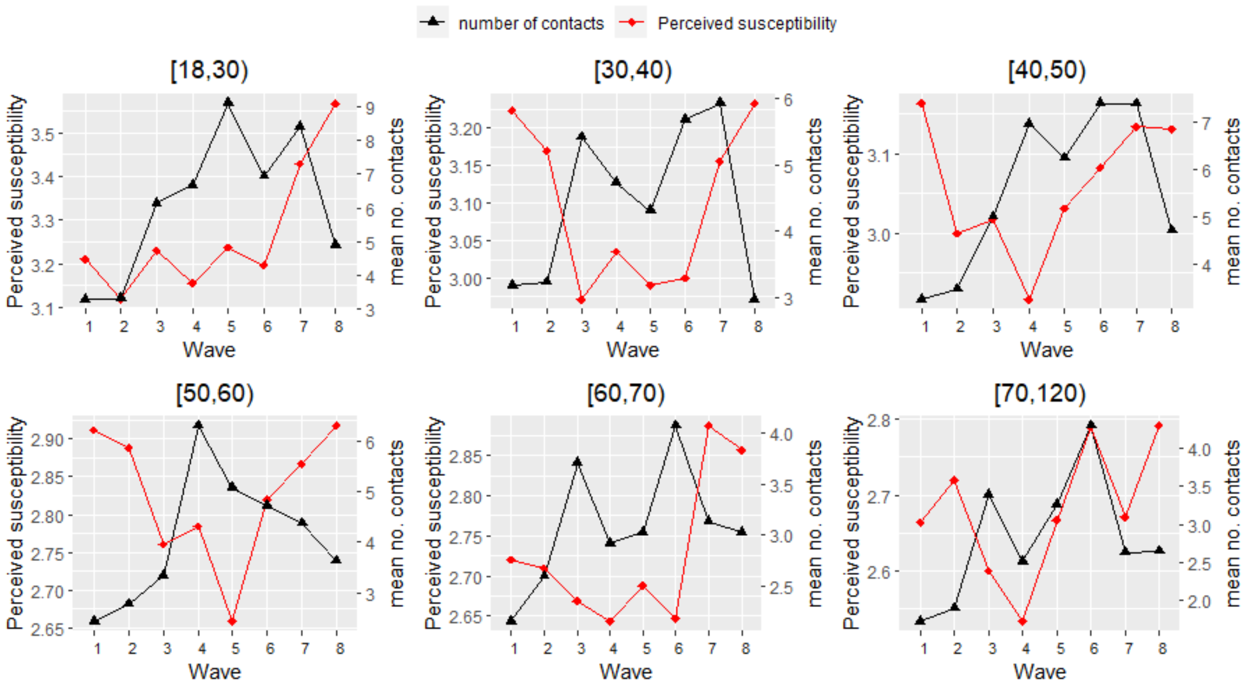

(b)

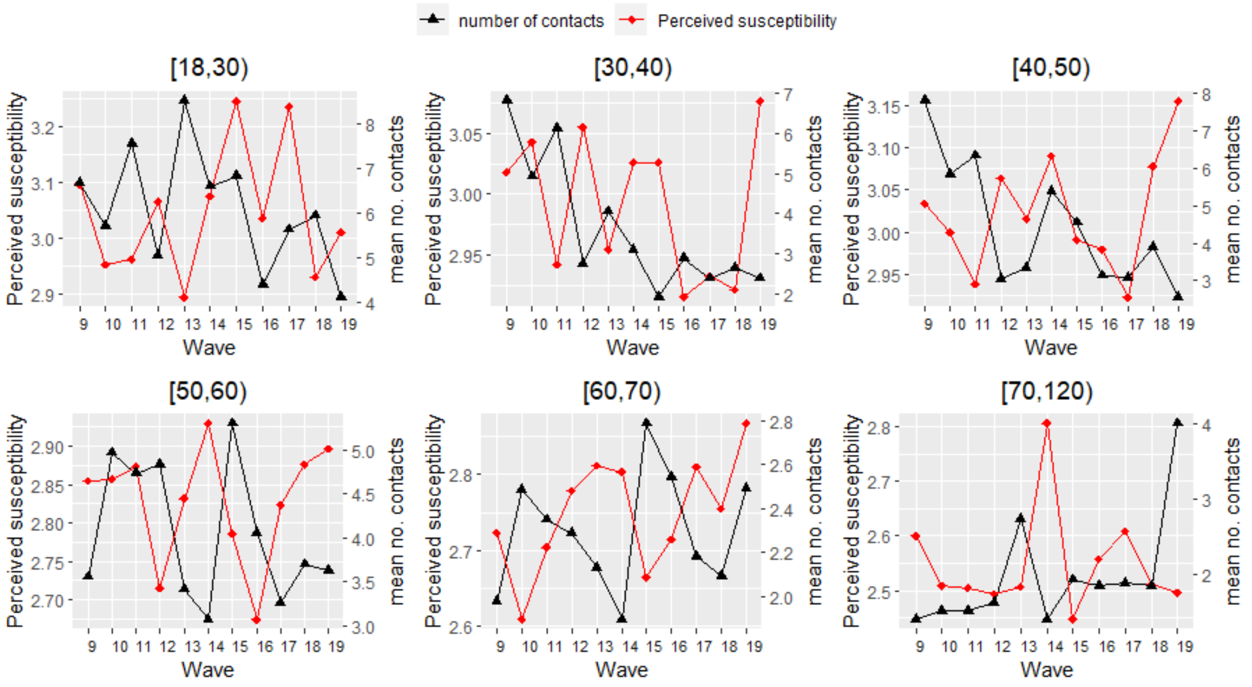

Figure S8: **(a):** Dynamics of perceived susceptibility and the number of social contacts in the first analysis. **(b):** Dynamics of perceived susceptibility and the number of social contacts in the second analysis. The lines show the mean score of the perceived susceptibility and average number of social contacts.

(a)

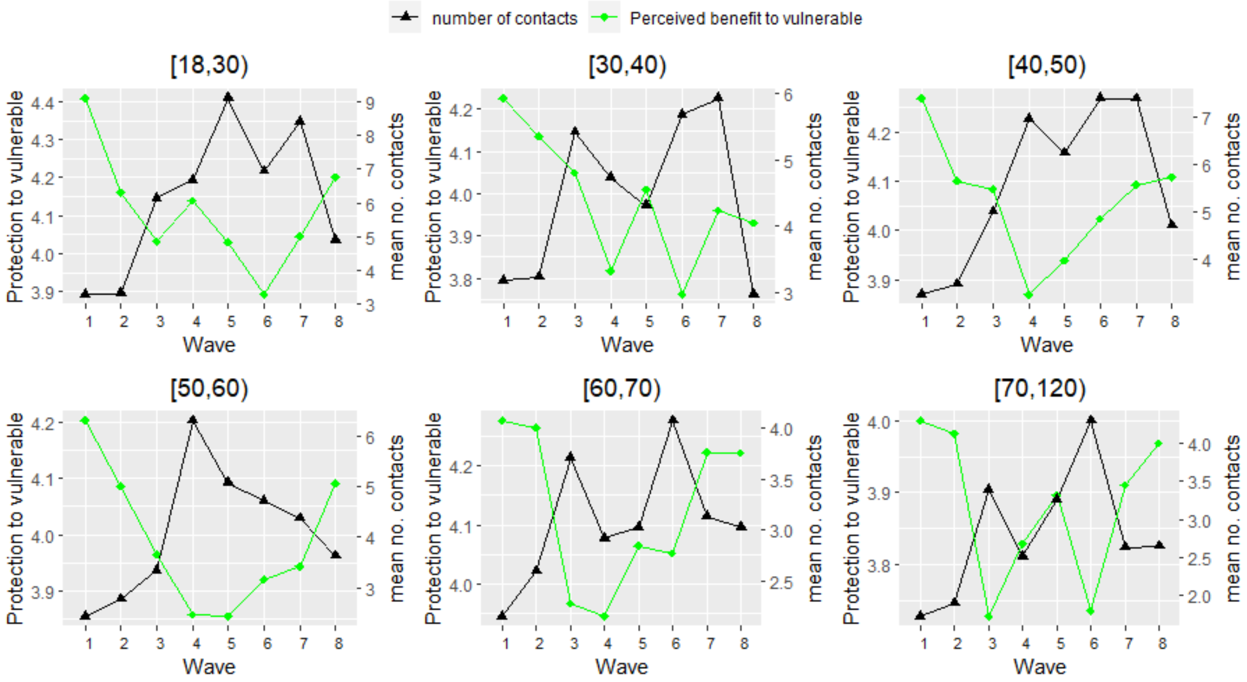

(b)

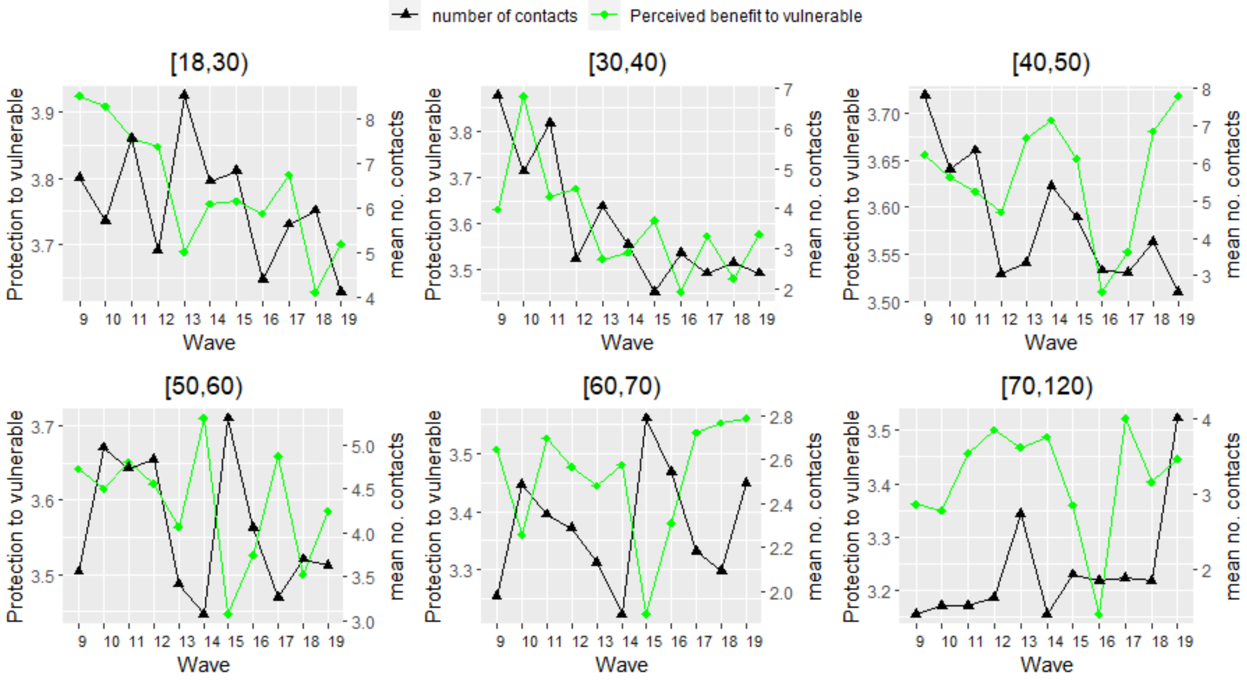

Figure S9: **(a):** Dynamics of perceived benefit to vulnerable and the number of social contacts in the first analysis. **(b):** Dynamics of perceived benefit to vulnerable and the number of social contacts in the second analysis. The lines show the mean score of the perceived benefit to vulnerable and average number of social contacts.

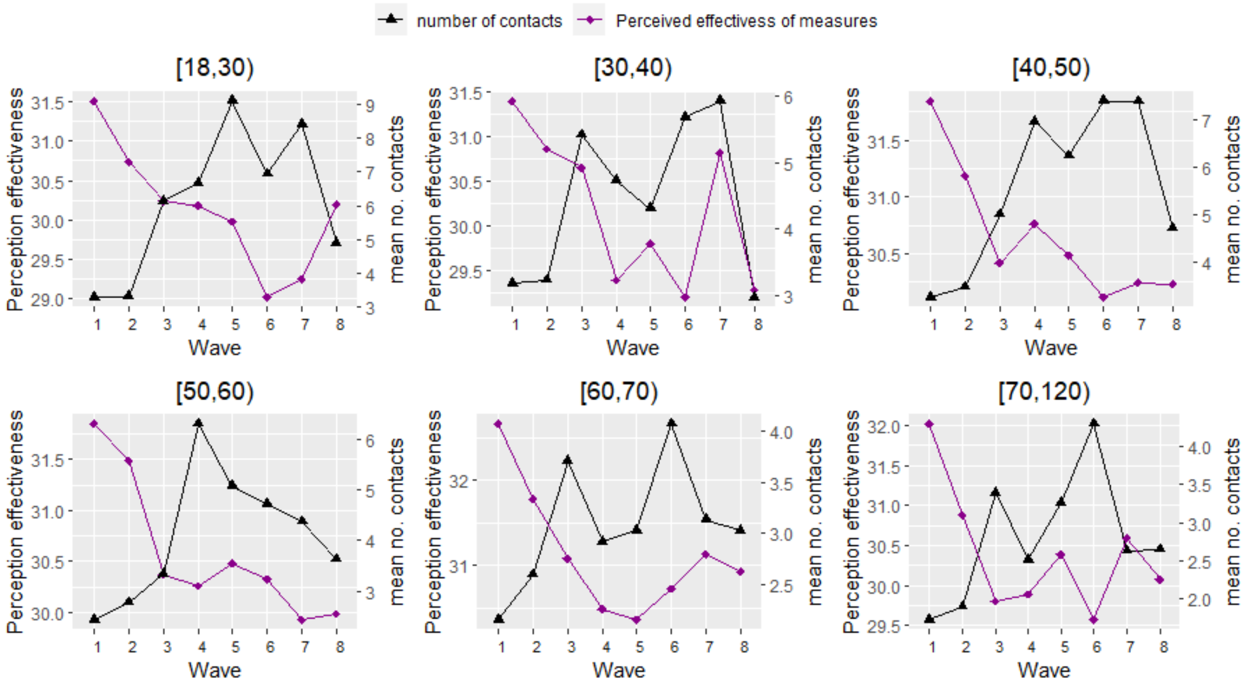

Figure S10: Dynamics of perceived effectiveness of measures and the number of social contacts. The lines show the mean score of the perceived effectiveness of measures and average number of social contacts for the first analysis.

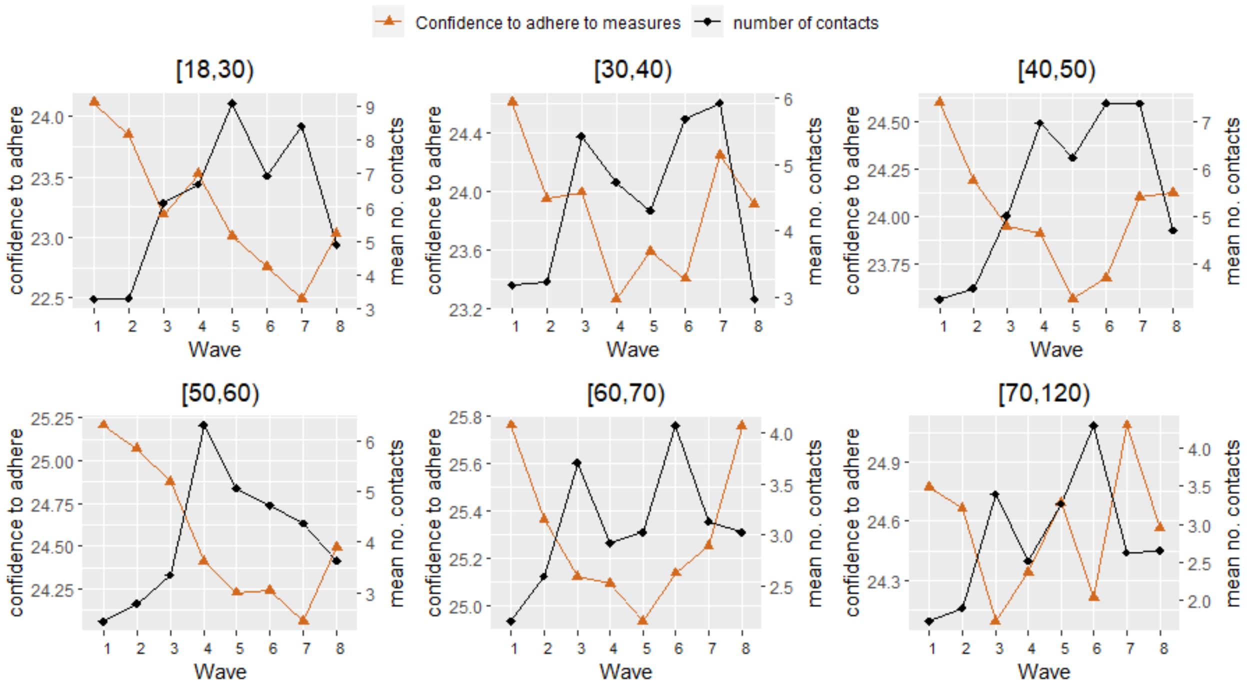

Figure S11: Dynamics of perceived adherence to measures and the number of social contacts. The lines show the mean score of the perceived adherence to measures and average number of social contacts for the first analysis.

(a)

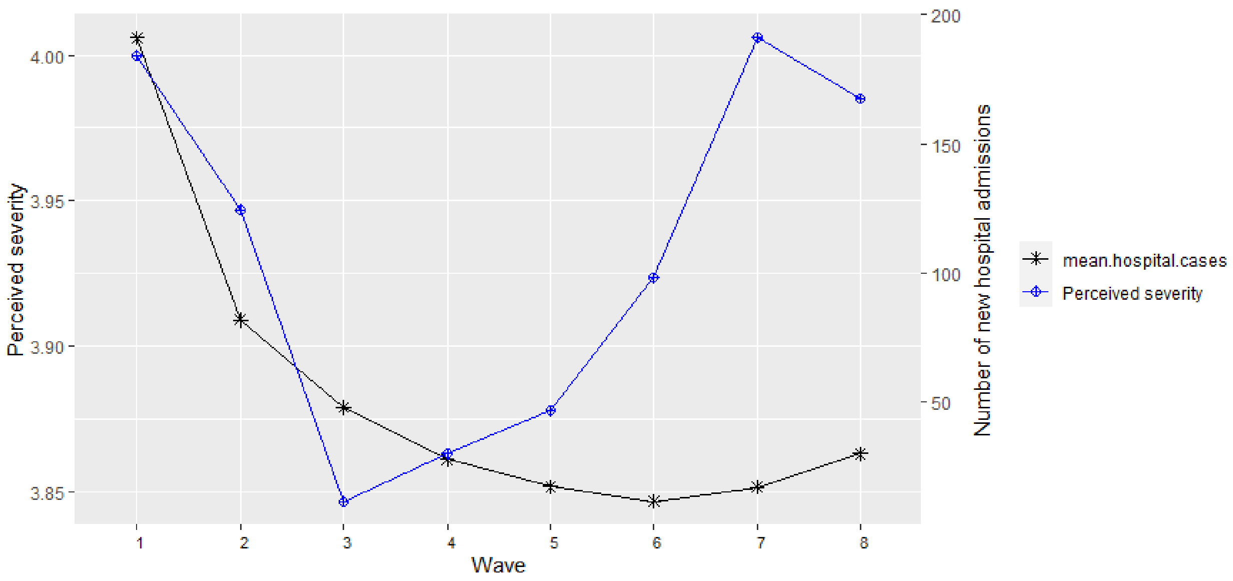

(b)

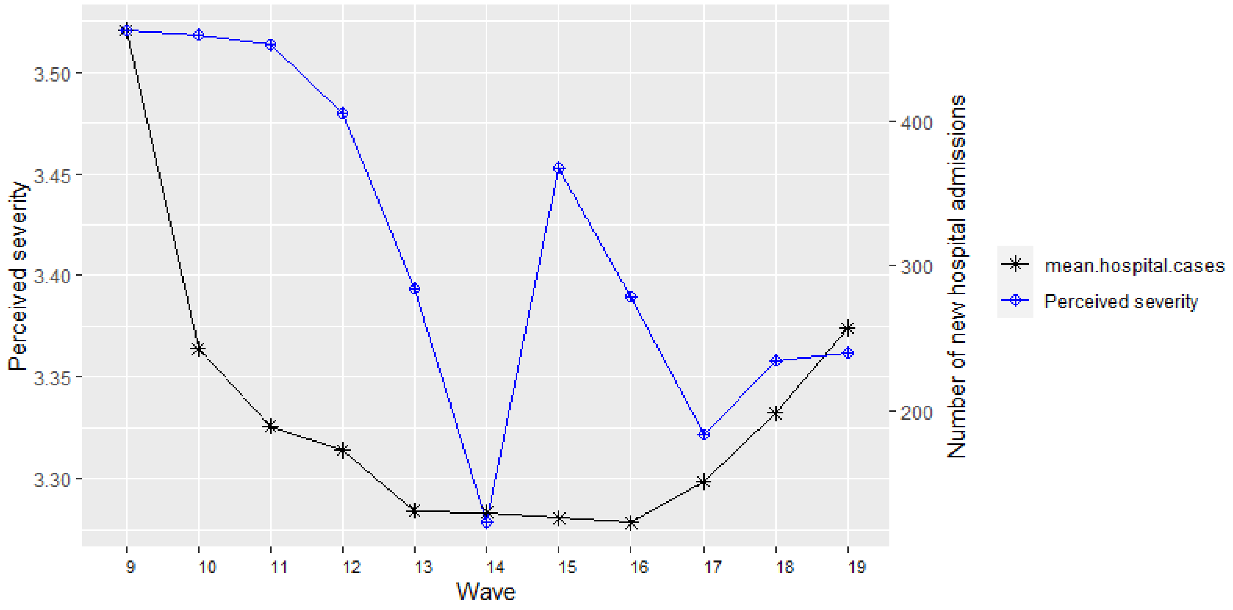

Figure S12: **(a):** Dynamics of perceived severity and the mean number of the reported new hospital admissions in the week of the survey and the preceding week in the first analysis. **(b):** Dynamics of perceived severity and the mean number of the reported new hospital admissions in the week of the survey and the preceding week in the second analysis. The lines show the mean score of the perceived severity and mean of the reported new hospital admissions.

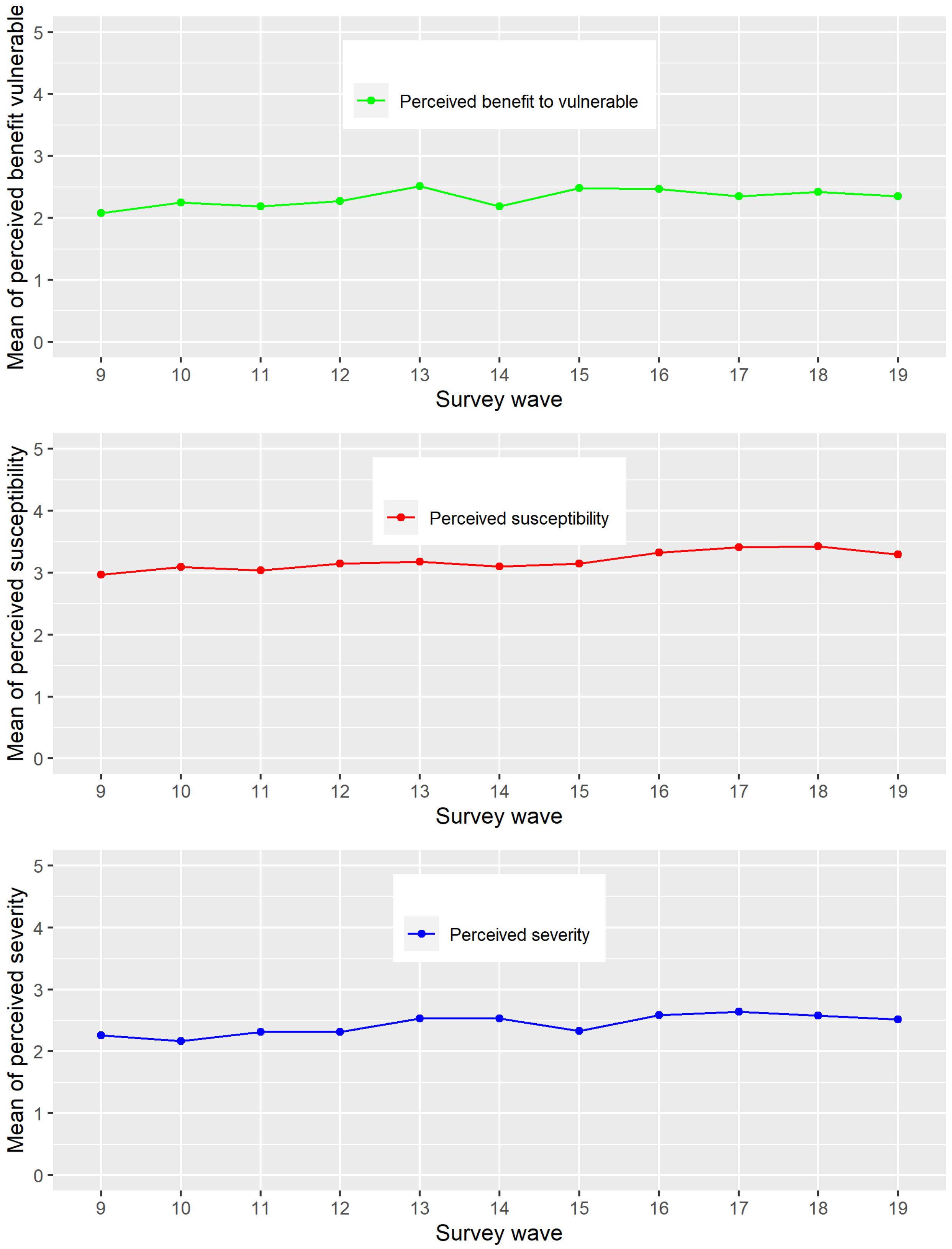

Figure S13: Plot of the average changes in perceived benefit to vulnerable, perceived susceptibility, and perceived severity for the vaccinated individuals before and after vaccination.

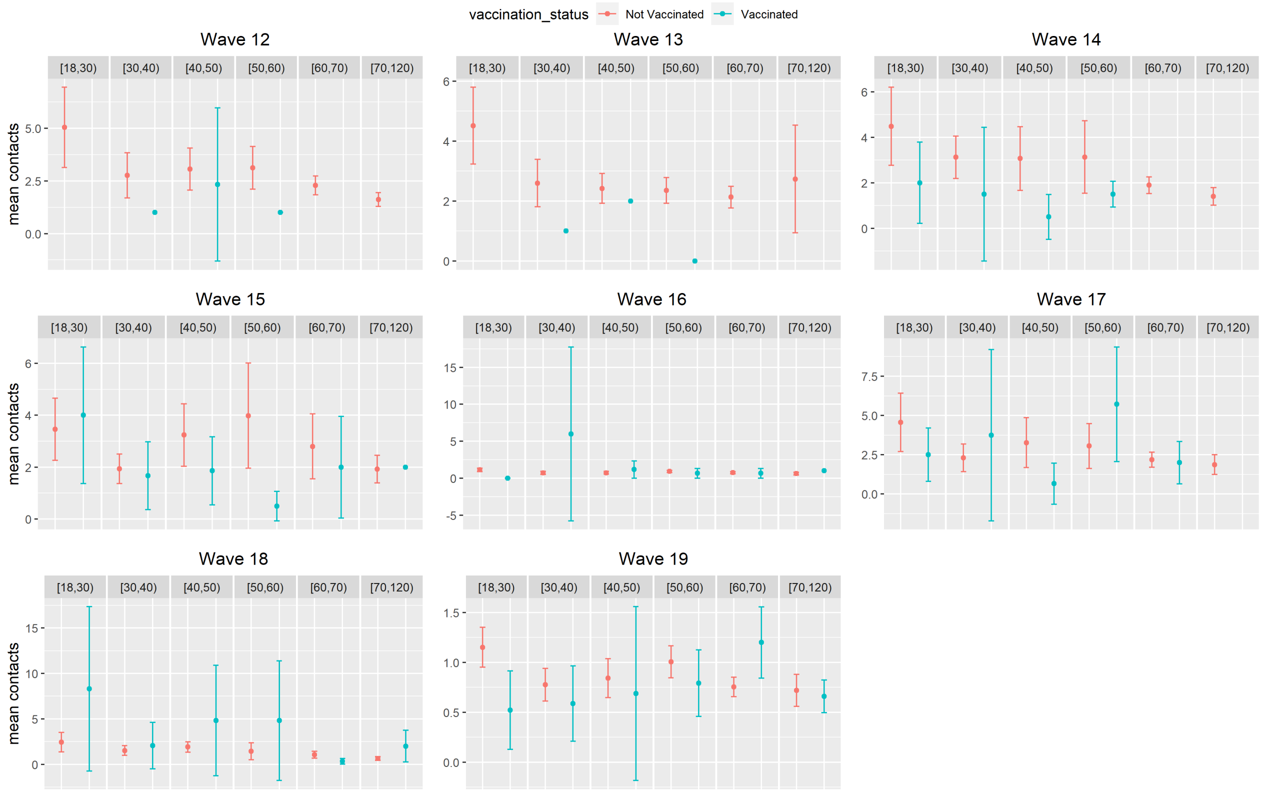

Figure S14: Plot of the average number of social contacts by vaccination status, wave of data collection and age group during the period when vaccines became available in the second part of the survey.

Age group	Wave 9 (1500) Mean (IQR)	Wave 10 (1340) Mean (IQR)	Wave 11 (1209) Mean (IQR)	Wave 12 (1087) Mean (IQR)	Wave 13 (983) Mean (IQR)	Wave 14 (883) Mean (IQR)	Wave 15 (799) Mean (IQR)	Wave 16 (719) Mean (IQR)	Wave 17 (645) Mean (IQR)	Wave 18 (1497) Mean (IQR)	Wave 19 (1383) Mean (IQR)
$[0, 5)$	-	-	-	7.58 (2-10)	11.84 (3-7)	14.11 (2-21)	13.17 (1-10)	6.45 (2-5)	11.66 (3-17)	9.00 (2-9)	5.04 (2-5)
$[5, 11)$	-	-	-	6.79 (2-4)	14.83 (3-24)	11.67 (3-14)	13.62 (3-19)	11.16 (2-12)	10.43 (3-16)	11.64 (3-14)	4.66 (2-4)
$[11, 18)$	-	-	-	4.43 (2-4)	10.35 (3-9)	7.60 (3-6)	8.40 (3-6)	4.38 (2-5)	7.64 (2-5)	7.71 (2-7)	4.44 (2-4)
$[18, 30)$	6.66 (1-5)	5.71 (1-5)	7.55 (1-5)	5.03 (1-5)	5.78 (1-4)	6.59 (1-4)	4.49 (1-4)	4.37 (1-4)	5.62 (1-5)	5.93 (1-4)	4.11 (1-4)
$[30, 40)$	6.80 (1-5)	4.91 (1-4)	6.12 (1-4)	2.75 (0.5-3)	4.05 (1-3)	3.09 (0-3)	1.91 (1-3)	2.87 (0-3)	2.37 (1-2)	2.63 (1-3)	2.38 (0-3)
$[40, 50)$	7.78 (1-5)	5.83 (1-4)	6.32 (1-4)	3.05 (0.5-3)	3.35 (1-3)	5.39 (0-3)	4.55 (0-4)	3.14 (1-3)	3.09 (1-3)	3.89 (0-4)	2.56 (1-3)
$[50, 60)$	3.56 (1-3)	4.96 (1-3)	4.73 (1-3)	3.09 (1-3)	3.41 (1-3)	3.07 (1-3)	5.29 (1-3)	4.05 (1-3)	3.26 (1-4)	3.70 (1-3)	2.64 (1-3)
$[60, 70)$	1.97 (1-2)	2.48 (1-2)	2.34 (1-2)	2.28 (1-2)	2.13 (1-3)	1.89 (1-2)	2.78 (1-3)	2.54 (1-3)	2.17 (1-3)	2.09 (1-2)	2.49 (1-2)
$[70, 120)$	1.39 (1-2)	1.51 (1-2)	1.50 (1-2)	1.62 (1-3)	2.73 (1-2)	1.40 (1-2)	1.92 (1-2)	1.84 (1-2)	1.84 (1-2)	1.83 (1-2)	1.71 (1-2)

Table S3: Average number of contacts in the second analysis.

Perceived severity Model (First analysis)			
Predictors	Incidence rate ratios	Confidence intervals (CI)	P-value
Intercept	0.90	0.69 - 1.16	0.405
Wave [1] - Ref	-	-	-
Wave [2]	1.01	0.90 - 1.13	0.865
Wave [3]	1.30	1.15 - 1.46	< 0.001
Wave [4]	1.42	1.26 - 1.61	< 0.001
Wave [5]	1.44	1.27 - 1.63	< 0.001
Wave [6]	1.63	1.44 - 1.84	< 0.001
Wave [7]	1.36	1.19 - 1.55	< 0.001
Wave [8]	1.09	0.95 - 1.24	< 0.001
household size	1.25	1.21 - 1.30	< 0.001
Gender[Male]	1.03	0.93 - 1.14	0.544
Day of week[weekend]	0.98	0.91 - 1.05	0.556
Face mask wearing [Yes]	1.33	1.24 - 1.42	<0.001
Occupation [Eight] - Ref	-	-	-
Occupation [One]	1.07	0.89 - 1.28	0.471
Occupation [Two]	1.31	0.95 - 1.79	0.097
Occupation [Three]	0.57	0.46 - 0.71	< 0.001
Occupation [Four]	0.99	0.79 - 1.25	0.938
Occupation [Five]	1.01	0.86 - 1.18	0.911
Occupation [Six]	0.14	0.05 - 0.43	< 0.001
Occupation [Seven]	0.84	0.50 - 1.40	0.497
Age group [[18,30)] - Ref	-	-	-
Age group [[30,40)]	1.39	1.07 - 1.80	0.014
Age group [[40,50)]	1.50	1.18 - 1.92	0.001
Age group [[50,60)]	1.32	1.03 - 1.68	0.026
Age group [[60,70)]	1.16	0.87 - 1.54	0.310
Age group [[70,120)]	1.15	0.83 - 1.58	0.407
Perceived severity [high perception] - Ref	-	-	-
Perceived severity [low perception]	1.70	1.18 - 2.44	0.004
Perceived severity [neutral perception]	1.56	1.14 - 2.14	0.005
wave [1]* Perceived severity [low perception] -Ref	-	-	-
wave [2]* Perceived severity [low perception]	0.90	0.64 - 1.26	0.538
wave [3]* Perceived severity [low perception]	0.83	0.60 - 1.14	0.246
wave [4]* Perceived severity [low perception]	0.70	0.49 - 0.98	0.038
wave [5]* Perceived severity [low perception]	0.93	0.64 - 1.33	0.679
wave [6]* Perceived severity [low perception]	0.54	0.37 - 0.79	0.002
wave [7]* Perceived severity [low perception]	1.18	0.80 - 1.72	0.402
wave [8]* Perceived severity [low perception]	0.79	0.53 - 1.18	0.251
wave [1]* Perceived severity [neutral perception] -Ref	-	-	-
wave [2]* Perceived severity [neutral perception]	1.08	0.83 - 1.40	0.557
wave [3]* Perceived severity [neutral perception]	0.80	0.61 - 1.03	0.088
wave [4]* Perceived severity [neutral perception]	1.08	0.84 - 1.40	0.557
wave [5]* Perceived severity [neutral perception]	0.86	0.66 - 1.12	0.261
wave [6]* Perceived severity [neutral perception]	0.76	0.58 - 0.99	0.045
wave [7]* Perceived severity [neutral perception]	0.84	0.63 - 1.13	0.247
wave [8]* Perceived severity [neutral perception]	0.73	0.55 - 0.97	0.032
Age group [[18,30)] * Perceived severity [low perception] - Ref	-	-	-
Age group [[30,40)] * Perceived severity [low perception]	0.61	0.41 - 0.91	0.017
Age group [[40,50)] * Perceived severity [low perception]	0.65	0.44 - 0.95	0.028
Age group [[50,60)] * Perceived severity [low perception]	0.71	0.46 - 1.10	0.127
Age group [[60,70)] * Perceived severity [low perception]	0.74	0.47 - 1.17	0.194
Age group [[70,120)] * Perceived severity [low perception]	0.64	0.37 - 1.11	0.115
Age group [[18,30)] * Perceived severity [neutral perception] -Ref	-	-	
Age group [[30,40)] * Perceived severity [neutral perception]	0.62	0.44 - 0.87	0.006
Age group [[40,50)] * Perceived severity [neutral perception]	0.58	0.42 - 0.81	0.001
Age group [[50,60)] * Perceived severity [neutral perception]	0.77	0.55 - 1.09	0.141
Age group [[60,70)] * Perceived severity [neutral perception]	0.68	0.48 - 0.96	0.031
Age group [[70,120)] * Perceived severity [neutral perception]	0.69	0.46 - 1.05	0.082

Table S4: Results for the first analysis (waves 1-8) from the generalized linear mixed effects model showing the incidence rate ratios, the associated confidence intervals and p-values for the perceived severity model.

Perceived severity Model (Second analysis)			
Predictors	Incidence rate ratios	Confidence intervals (CI)	P-value
Intercept	0.68	0.49 - 0.90	0.010
Wave [9] - Ref	-	-	-
Wave [10]	0.88	0.76 - 1.02	0.079
Wave [11]	0.89	0.77 - 1.03	0.105
Wave [12]	0.83	0.71 - 0.97	0.022
Wave [13]	0.86	0.73 - 1.01	0.071
Wave [14]	0.70	0.58 - 0.84	< 0.001
Wave [15]	0.82	0.69 - 0.97	0.020
Wave [16]	0.87	0.72 - 1.04	0.131
Wave [17]	0.76	0.63 - 0.93	0.007
Wave [18]	0.87	0.75 - 1.00	0.056
Wave [19]	0.76	0.65 - 0.88	<0.001
household size	1.45	1.38 - 1.55	< 0.001
Gender[Male]	0.88	0.75 - 0.90	0.008
Day of week[weekend]	1.01	0.92 - 1.11	0.842
Occupation [Eight] - Ref	-	-	-
Occupation [One]	1.19	0.96 - 1.48	0.105
Occupation [Two]	1.13	0.78 - 1.65	0.510
Occupation [Three]	0.88	0.72 - 1.09	0.240
Occupation [Four]	0.99	0.78 - 1.25	0.931
Occupation [Five]	1.83	1.53 - 2.19	< 0.001
Occupation [Six]	0.56	0.22 - 1.45	0.226
Occupation [Seven]	1.10	0.72 - 1.69	0.664
Age group [[18,30)] - Ref	-	-	-
Age group [[30,40)]	0.87	0.64 - 1.21	0.443
Age group [[40,50)]	0.95	0.71 - 1.25	0.747
Age group [[50,60)]	0.97	0.72 - 1.30	0.817
Age group [[60,70)]	0.96	0.70 - 1.31	0.802
Age group [[70,120)]	0.82	0.58 - 1.17	0.280
High risk status [No] - Ref	-	-	-
High risk status [Yes]	1.03	0.93 - 1.13	0.585
Face mask wearing [No] - Ref	-	-	-
Face mask wearing [Yes]	1.93	1.78 - 2.08	< 0.001
Perceived severity [high perception] - Ref	-	-	-
Perceived severity [low perception]	1.62	1.14 - 2.31	0.007
Perceived severity [neutral perception]	1.76	1.31 - 2.38	< 0.001
Age group [[18,30)] * Perceived severity [low perception] - Ref	-	-	-
Age group [[30,40)] * Perceived severity [low perception]	0.85	0.59- 1.23	0.395
Age group [[40,50)] * Perceived severity [low perception]	0.74	0.51 - 1.07	0.108
Age group [[50,60)] * Perceived severity [low perception]	0.81	0.55 - 1.18	0.273
Age group [[60,70)] * Perceived severity [low perception]	0.72	0.49 - 1.06	0.093
Age group [[70,120)] * Perceived severity [low perception]	0.52	0.29 - 0.95	0.032
Age group [[18,30)] * Perceived severity [neutral perception] -Ref	-	-	
Age group [[30,40)] * Perceived severity [neutral perception]	0.84	0.60 - 1.20	0.342
Age group [[40,50)] * Perceived severity [neutral perception]	0.98	0.71 - 1.35	0.884
Age group [[50,60)] * Perceived severity [neutral perception]	0.70	0.51 - 0.96	0.028
Age group [[60,70)] * Perceived severity [neutral perception]	0.79	0.58 - 1.09	0.148
Age group [[70,120)] * Perceived severity [neutral perception]	0.75	0.51 - 1.12	0.161
wave [9]* Perceived severity [low perception] -Ref	-	-	-
wave [10]* Perceived severity [low perception]	1.25	0.91 - 1.72	0.161
wave [11]* Perceived severity [low perception]	1.16	0.85 - 1.57	0.351
wave [12]* Perceived severity [low perception]	1.06	0.75 - 1.48	0.754
wave [13]* Perceived severity [low perception]	0.94	0.67 - 1.33	0.730
wave [14]* Perceived severity [low perception]	1.13	0.80 - 1.60	0.473
wave [15]* Perceived severity [low perception]	0.70	0.48 - 1.01	0.055
wave [16]* Perceived severity [low perception]	0.97	0.67 - 1.41	0.873
wave [17]* Perceived severity [low perception]	0.76	0.52 - 1.11	0.159
wave [18]* Perceived severity [low perception]	0.63	0.46 - 0.86	0.003
wave [19]* Perceived severity [low perception]	0.74	0.54 - 1.01	0.059
wave [9]* Perceived severity [neutral perception] -Ref	-	-	-
wave [10]* Perceived severity [neutral perception]	0.84	0.66 - 1.07	0.166
wave [11]* Perceived severity [neutral perception]	1.01	0.79 - 1.30	0.907
wave [12]* Perceived severity [neutral perception]	0.62	0.47 - 0.83	0.001
wave [13]* Perceived severity [neutral perception]	0.63	0.47 - 0.84	0.002
wave [14]* Perceived severity [neutral perception]	0.78	0.58 - 1.04	0.088
wave [15]* Perceived severity [neutral perception]	0.67	0.49 - 0.91	0.010
wave [16]* Perceived severity [neutral perception]	0.60	0.44 - 0.83	0.002
wave [17]* Perceived severity [neutral perception]	0.59	0.42 - 0.82	0.002
wave [18]* Perceived severity [neutral perception]	0.66	0.52 - 0.85	0.001
wave [19]* Perceived severity [neutral perception]	0.61	0.47 - 0.79	< 0.001

Table S5: Results for the second analysis (waves 9-19) from the generalized linear mixed effects model showing the incidence rate ratios, the associated confidence intervals and p-values for the perceived severity model.

Perceived severity Model (First analysis)			
Variable	Chisq	Df	Pr (> chisq)
Intercept	0.694	1	0.404
Wave	103.187	7	<0.001
Household size	131.380	1	< 0.001
Gender	0.369	1	0.544
Occupation	53.062	7	< 0.001
Face mask wearing	71.417	1	<0.001
High risk status	0.003	1	0.954
Age group	13.201	5	0.021
Perceived severity	12.017	2	0.002
Day of week	0.346	1	0.556
Wave : Perceived severity	35.812	14	0.002
Age group : Perceived severity	15.452	10	0.116
Perceived severity Model (Second analysis)			
Variable	Chisq	Df	Pr (> chisq)
Intercept	5.766	1	0.016
Wave	23.130	10	0.010
Household size	156.124	1	< 0.001
Gender	4.437	1	0.035
Occupation	73.988	7	< 0.001
Face mask wearing	275.936	1	< 0.001
Age group	3.144	5	0.682
High risk status	0.297	1	0.585
Perceived severity	14.761	2	< 0.001
Day of week	0.039	1	0.842
Wave : Perceived severity	66.585	20	< 0.001
Age group : Perceived severity	16.092	10	0.039

Table S6: Analysis of Deviance Table (Type III Wald tests) for both the first analysis (waves 1-8) and second analysis (waves 9-19) for the perceived severity models.

Perceived susceptibility Model (First analysis)			
Variable	Chisq	Df	Pr (> chisq)
Intercept	1.574	1	0.209
Wave	33.773	7	< 0.001
Household size	30.282	1	< 0.001
Gender	0.491	1	0.483
Occupation	50.806	7	< 0.001
Face mask wearing	27.708	1	< 0.001
Age group	9.313	5	0.097
High risk status	0.009	1	0.969
Perceived susceptibility	4.930	2	0.085
Day of week	0.502	1	0.478
Face mask wearing : Perceived susceptibility	5.748	2	0.05
Household : Perceived susceptibility	8.535	2	0.014
Wave : Perceived susceptibility	24.658	14	0.038
Age group : Perceived susceptibility	20.435	10	0.025
Perceived susceptibility Model (Second analysis)			
Variable	Chisq	Df	Pr (> chisq)
Intercept	0.186	1	0.666
Wave	109.190	10	< 0.001
Household size	162.083	1	< 0.001
Gender	4.360	1	0.036
Occupation	71.539	7	< 0.001
Face mask wearing	285.047	7	< 0.001
Age group	3.498	5	0.623
High risk status	0.009	1	0.924
Perceived susceptibility	21.039	2	< 0.001
Day of week	0.646	1	0.421
Wave : Perceived susceptibility	55.266	20	< 0.001
Age group : Perceived susceptibility	36.134	10	< 0.001

Table S7: Analysis of Deviance Table (Type III Wald tests) for both the first analysis (waves 1-8) and second analysis (waves 9-19) for the perceived susceptibility models.

Perceived benefit to vulnerable model Model (First analysis)			
Variable	Chisq	Df	Pr (> chisq)
Intercept	0.565	1	0.452
Wave	98.733	7	< 0.001
Household size	132.791	1	< 0.001
Gender	0.481	1	0.488
Occupation	50.076	7	< 0.001
Face mask wearing	70.191	1	<0.001
High risk status	0.001	1	0.996
Age group	5.643	5	0.342
Perceived benefit to vulnerable	0.361	2	0.834
Day of week	0.037	1	0.846
Wave : Perceived benefit to vulnerable	26.005	14	0.025
Weekday : Perceived benefit to vulnerable	7.267	2	0.026
Perceived benefit to vulnerable (Second analysis)			
Variable	Chisq	Df	Pr (> chisq)
Intercept	0.194	1	0.659
Wave	101.370	10	< 0.001
Household size	155.673	1	< 0.001
Gender	3.989	1	0.045
Occupation	76.184	7	< 0.001
face mask wearing	279.765	7	< 0.001
Age group	11.803	5	0.037
Perceived benefit to vulnerable	9.992	2	0.006
High risk status	0.001	1	0.994
Day of week	1.204	1	0.273
Wave : Perceived benefit to vulnerable	30.340	20	0.05

Table S8: Analysis of Deviance Table (Type III Wald tests) for both the first analysis (waves 1-8) and second analysis (waves 9-19) for the Perceived benefit to vulnerable models.

Perceived effectiveness of measures Model (First analysis)			
Variable	Chisq	Df	Pr (> chisq)
Intercept	1.665	1	0.196
Wave	24.310	7	0.001
Household size	133.061	1	< 0.001
Gender	0.669	1	0.413
Occupation	51.491	7	< 0.001
Face mask wearing	72.848	1	<0.001
High risk status	0.054	1	0.816
Age group	5.594	5	0.348
Perceived effectiveness of measures	1.097	1	0.295
Day of week	0.595	1	0.440
Wave : Perceived effectiveness of measures	17.658	7	0.013
Perceived adherence to measures Model (First analysis)			
Variable	Chisq	Df	Pr (> chisq)
Intercept	4.390	1	0.036
Wave	113.119	7	< 0.001
Household size	132.119	1	< 0.001
Gender	0.713	1	0.398
Occupation	52.191	7	< 0.001
Face mask wearing	71.201	1	<0.001
High risk status	0.123	1	0.726
Age group	14.626	5	0.012
Perceived adherence to measures	6.013	1	0.014
Day of week	0.552	1	0.457
Age group : Perceived adherence to measures	13.965	5	0.015

Table S9: Analysis of Deviance Table (Type III Wald tests) for the first analysis (waves 1-8) for the perceived effectiveness of measures and perceived adherence to measures models.

Participants occupation categories	Occupations
Category One	craftsman, trader with 5 employees or less<br>industrial, wholesaler with 6 employees or more<br>member of the general management, senior executive responsible for 11 employees or more<br>member of the general management, senior executive responsible for 6 to 10 employees<br>member of the general management, senior executive responsible for 5 employees or less<br>middle management, that is not part of the general management, responsible for 5 employees or less<br>middle management, that is not part of the general management, responsible for 6 employees or more
Category Two	farmer<br>liberal profession or profession for which qualification is required
Category Three	house man or housewife<br>never worked<br>unable for work<br>unemployed
Category Four	pre-retired<br>retired
Category Five	non-skilled worker<br>skilled worker<br>other employee who does not do office work (eg teacher, nurses ...)
Category Six	other
Category Seven	student
Category Eight	other employee who mainly performs office work

Table S10: Categories of participants' occupation grouped according to similarity of professions.
